# Co-Inhibition of tGLI1 and GP130 Using FDA-Approved Ketoconazole and Bazedoxifene Is Synergistic Against the Growth and Metastasis of HER2-Enriched and Triple-Negative Breast Cancers

**DOI:** 10.3390/cells13242087

**Published:** 2024-12-17

**Authors:** Sara Manore, Chuling Zhuang, Mariana K. Najjar, Grace L. Wong, Shivani Bindal, Kounosuke Watabe, Jiayuh Lin, Hui-Wen Lo

**Affiliations:** 1 Vivian L. Smith Department of Neurosurgery, McGovern Medical School, The University of Texas Health Science Center at Houston, Houston, TX 77030, USA; manores2@winthropalumni.com (S.M.); chuling.zhuang@uth.tmc.edu (C.Z.); mariana.k.najjar@uth.tmc.edu (M.K.N.); glwong@houstonmethodist.org (G.L.W.); shivani.bindal@uth.tmc.edu (S.B.); 2 Wake Forest Graduate School of Biomedical Sciences, Wake Forest University School of Medicine, Winston-Salem, NC 27101, USA; 3 Graduate School of Biomedical Sciences, The University of Texas Health Science Center at Houston, Houston, TX 77030, USA; 4 Department of Cancer Biology, Wake Forest University School of Medicine, Winston-Salem, NC 27101, USA; kwatabe@wakehealth.edu; 5 Department of Biochemistry and Molecular Biology, University of Maryland School of Medicine, Baltimore, MD 21201, USA; jlin@som.umaryland.edu

**Keywords:** tGLI1, STAT3, IL-6, GP130, ketoconazole, bazedoxifene, combination therapy, breast CSCs, TNBC, HER2-enriched breast cancer

## Abstract

Breast cancer stem cells (CSCs) are resistant to most cancer therapeutics and contribute to tumor recurrence and metastasis. Two breast CSC-promoting transcription factors, truncated glioma-associated oncogene homolog 1 (tGLI1) and signal transducer and activator of transcription 3 (STAT3), have been reported to be frequently co-expressed in HER2-enriched breast cancer and triple-negative breast cancer (TNBC), undergo protein-protein interactions for gene regulation and activation, and functionally cooperate to promote breast CSCs. STAT3 can be activated by activated interleukin-6 receptor/glycoprotein-130 (IL-6R/GP130). Co-targeting of tGLI1 and IL-6R/GP130 has not been investigated in breast cancer or any tumor type. Here, we report that tGLI1 and GP130 are co-overexpressed in the majority of HER2-enriched breast cancers and TNBCs at 53.8% and 44.4%, respectively. tGLI1+IL-6/IL-6R/GP130 signaling is frequently co-enriched and co-activated in HER2-enriched breast cancer and TNBC when compared to luminal subtypes. tGLI1+GP130 co-overexpression strongly promotes CSCs of HER2-enriched breast cancer and TNBC. FDA-approved tGLI1 inhibitor Ketoconazole and GP130 inhibitor Bazedoxifene synergize against breast cancer proliferation and CSC phenotypes in vitro and reduce TNBC tumor growth and metastatic burden in vivo. Our study demonstrates, for the first time, that co-targeting tGLI1 and IL-6R/GP130/STAT3 signaling pathways is synergistic against HER2-enriched breast cancer and TNBC, warranting future clinical investigations.

## 1. Introduction

Breast cancer is the most commonly diagnosed cancer in American women and the second most common cause of cancer-related deaths [1]. Breast cancer can be categorized into molecular subtypes based on estrogen receptor (ER), progesterone receptor (PR), and human epidermal growth factor receptor 2 (HER2) expression, namely, Luminal A, Luminal B, HER2-enriched, and triple-negative breast cancer (TNBC) [2]. Luminal A (ER+/PR+, HER2-) is the most common and least aggressive breast cancer subtype and often responds to hormonal-based therapies, attributing to better clinical outcomes [3,4]. Luminal B tumors (ER+/PR+) can be HER2+ or HER2-, whereas the HER2-enriched breast cancer subtype lacks ER/PR expression. Standard-of-care (SOC) management for HER2-positive breast cancers includes surgical resection and HER2-targeted therapies that may be combined with chemotherapy [5]. However, despite efficacious treatment options, HER2-enriched breast tumors are often associated with therapeutic resistance and tumor relapse [6,7]. Triple-negative breast carcinomas lack ER, PR, and HER2 expression, rendering this subtype difficult to treat [8].

In this study, we exploit two signaling pathways frequently hyperactivated in breast cancer, truncated glioma-associated oncogene homolog 1 (tGLI1) and interleukin-6/interleukin-6 receptor/glycoprotein 130 (IL-6/IL-6R/GP130) signaling, to directly target HER2-enriched breast cancer and TNBC. TGLI1 is an alternatively spliced variant of the GLI1 transcription factor and is a terminal effector in the Sonic Hedgehog pathway [9]. Despite the lack of exon III and partial loss of exon IV, tGLI1 retains all GLI1 functional domains and is a gain-of-function oncogenic transcription factor [9,10,11,12,13,14]. Interestingly, tGLI1, but not GLI1, is expressed in a tumor-specific manner in both glioblastoma and breast cancer, rendering tGLI1 an ideal therapeutic target. We recently identified a Food and Drug Administration (FDA)-approved antifungal, ketoconazole (Nizoral^®^; KCZ), as the first inhibitor to selectively target tGLI1-positive breast cancer cells, directly bind tGLI1, and antagonize tGLI1 transcriptional activity [12]. Additionally, tGLI1 can functionally interact with signal transducer and activator of transcription 3 (STAT3) in breast cancer. TGLI1 and STAT3 are concurrently activated in HER2-enriched breast cancer and TNBC subtypes. This co-activation promotes breast cancer stem cell (CSC) phenotypes and presents a novel combination therapy for the clinic [13]. In the present study, we identified a novel combinatorial therapy through pharmacological inhibition of tGLI1 and GP130, an upstream regulator of STAT3, in HER2-enriched breast cancer and TNBC.

IL-6/IL-6R/GP130 signaling is responsible for innate and acquired immune responses but is frequently hijacked in multiple cancer types to promote anti-immune evasion and a pro-inflammatory tumor microenvironment [15]. The IL-6 cytokine is upregulated in sera of breast cancer patients when compared to normal samples [16,17]. Dysregulated IL-6/IL-6R/GP130 signaling promotes breast cancer cell proliferation, breast CSCs, and therapeutic resistance, contributing to tumor recurrence [18,19,20,21,22,23,24,25]. IL-6/IL-6R/GP130 signaling starts when IL-6 binds IL-6Rα, creating a high affinity for the membrane-bound GP130 receptor [26]. Two trimeric complexes homodimerize and, in turn, recruit Janus kinases (JAKs) to phosphorylate and activate STAT3 for gene regulation [27,28,29,30]. In the present study, we used Bazedoxifene (DUAVEE^®^; BZA), which directly binds the GP130 receptor to inhibit IL-6-induced STAT3 activation in breast cancer [31,32]. Administered with conjugated estrogens, BZA is an FDA-approved selective estrogen receptor modulator (SERM) for the treatment of post-menopausal osteoporosis [33]. In this study, we identified tGLI1 and IL-6/IL-6R/GP130 signaling pathways to be frequently co-overexpressed, co-activated, and functionally cooperate to promote breast CSCs in HER2-enriched breast cancer and TNBC. Furthermore, we identified a novel combinatorial treatment using KCZ and BZA, which synergizes to reduce cell viability of HER2-enriched breast cancer cells and TNBC cells, suppress breast CSCs, decrease basal primary breast tumor growth using a patient-derived xenograft (PDX) model, and reduce metastatic burden using a TNBC intracardiac mouse model. Collectively, our findings demonstrate that dual-targeting tGLI1 and IL-6/IL-6R/GP130 signaling pathways are effective against HER2-enriched breast cancer and TNBC and support the use of tGLI1 and IL-6/IL-6R/GP130 inhibitors for the treatment of these more aggressive subtypes of breast cancer.

## 2. Materials and Methods

### 2.1. Cell Lines and Reagents

HMEC, T47D, MCF-7, MCF10A, BT-20, and MDA-MB-468 human breast cell lines were purchased from the American Type Culture Collection (ATCC) (Manassas, VA, USA) and cultured according to ATCC recommendations. JIMT-1 and SUM-159 human breast cancer cells were purchased from the core facility at the Wake Forest School of Medicine (WFSM). The MDA-MB-231 cell line was obtained from the Massagué laboratory and cultured in Dulbecco’s Modified Eagle Medium (DMEM) supplemented with 10% fetal bovine serum (FBS, Corning 35-10-CV) (Corning, NY, USA) and 1% penicillin–streptomycin solution (P/S, Corning 30-002-CI) [34]. Trastuzumab-resistant BT474 (BT474-TzmR) and BT474 breast cancer cell lines were graciously given to our lab by Dr. Dihua Yu [35]. Brain-tropic SKBRM breast cancer cells, a variant of HER2-enriched SKBR3, were a kind gift from Drs. Fei Xing and Kounosuke Watabe [36]. Isogenic MDA-MB-231 and SKBRM breast cancer cell lines with stable overexpression of vector or tGLI1 were previously described [10]. The E6/E7/hTERT immortalized human astrocyte cell line was graciously gifted by Dr. Russell Pieper (University of California-San Francisco). All cell lines were routinely tested for mycoplasma contamination; if detected, cells were treated with BM-Cyclin (Sigma-Aldrich 10-799-050-001) (St. Louis, MO, USA) and tested again prior to use. Plasmids pCMV-Tag2b-Vector and pCMV-Tag2b-tGLI1 were used for transient transfection and were previously generated by our laboratory [9]. The IL6ST (HG10974-NM) overexpression plasmid and its respective negative control were purchased from Sino Biological (Beijing, China). Ketoconazole was purchased from Cayman Chemical (1512) (Ann Arbor, MI, USA), and Bazedoxifene acetate was obtained from AdooQ Bioscience (A11665) (Irvine, CA, USA). Stock solutions were prepared using 100% dimethyl sulfoxide (DMSO) for cell culture use.

### 2.2. Metastasis-Free Survival (MFS), Gene Set Enrichment Analyses (GSEA), and Statistical Analysis

For data mining, publicly available RNA-seq data sets of breast cancer patients were obtained from The Cancer Genome Atlas (TCGA) and Gene Expression Omnibus (GEO) (GSE12276/2034/2603/5327/14020). Median-centered scores were derived to generate a combined tGLI1 and IL-6/IL-6R/GP130 activation signature (tGAS+IL-6/IL-6R/GP130). The tGLI1 activation signature (TGAS) was generated in our lab and is comprised of validated tGLI1 target genes regulated by tGLI1 but not GLI1 [14]. The IL-6/IL-6R/GP130 activation signature contains IL-6/IL-6R/GP130 and STAT3 target genes [37,38,39]. Combined tGAS+IL-6/IL-6R/GP130 activation scores obtained from TCGA or GEO datasets were stratified by breast cancer subtype. ANOVA and Tukey’s post-hoc analysis were performed to calculate statistical significance using GraphPad Prism 8 (GraphPad Prism 9.0 Software) (San Diego, CA, USA). GSEA analyses were conducted as previously described [13]. The Kaplan-Meier MFS plot of breast cancer patients was generated using the GEO database through high vs. low stratification of tGAS and IL-6/IL-6R/GP130 activation scores. A log-rank test was used for statistical significance.

### 2.3. Cell Viability Assay and Combination Index (CI) Analyses

Cell-based viability assays were performed, and combination indices (CI) were calculated as previously described [9]. CIs were derived using the Chou–Talalay method, which defines drug combinations as either additive (CI = 1), synergistic (CI < 1), or antagonistic (CI > 1) [40]. ANOVA and Tukey’s post-hoc analyses were performed to calculate statistical significance.

### 2.4. Western Blotting

Western blotting was conducted as previously described [12]. Antibodies used include GLI1 (Cell Signaling Technology/CST 2643, 1:1000) (Danvers, MA, USA), a custom-made tGLI1-specific antibody (YenZym, 1:1000) (Brisbane, CA, USA) previously validated in our lab [13], GP130 (CST 3732, 1:1000), pSTAT3 (CST 9131L, 1:1000), STAT3 (CST 9139, 1:1000), OCT4 (CST 2750 and CST 4286, 1:1000), Nanog (CST 4903, 1:1000), SOX2 (CST 4900, 1:1000), Cyclin D1 (CST 2922, 1:1000), Bcl2 (CST 4223, 1:1000), BclXL (CST 2764, 1:1000), Cep70 (Proteintech 16280-1-AP, 1:1000) (Rosemont, IL, USA), UPF3A (Proteintech 17114-1-AP, 1:1000), RRas2 (Proteintech 12530-1-AP, 1:1000), PARP (CST 9532), α-Tubulin (Sigma-Aldrich T6074, 1:5000–10,000), β-actin (CST 3700, 1:5000–10,000), and Vinculin (CST 13901, 1:5000–10,000).

### 2.5. Scratch-Wound Assay

Briefly, cells were seeded at 5–7.5 × 10^5^ cells/well in a 6-well culture dish to reach 80–90% confluency 24 h later. Cells were then scratched using a p200 pipette tip, and media was replaced to remove floating cells. Time 0 images were obtained using the KEYENCE cell imaging system (Itasca, IL, USA). Treatment media was subsequently added to respective wells. Cells were reimaged 24 h later. ImageJ was used for analysis, and migrating cells were quantified as previously described [41].

### 2.6. Animal Studies

All mice were housed in a pathogen-free facility at the Animal Research Program at WFSM under a 12/12 h light/dark cycle and fed irradiated rodent chow ad libitum. The animal handling procedures were approved by the Institutional Animal Care and Use Committee (IACUC) at WFSM. TNBC patient-derived xenograft (PDX)-3887 was graciously given to our lab by Michael T. Lewis, Ph.D., at the Baylor College of Medicine [42]. We serially maintain PDXs by surgically inoculating them into the mammary fat pad (MFP) as previously described [14]. The left inguinal mammary fat pad (MFP) of 4–5 week-old female athymic mice (Charles River) (Wilmington, MA, USA) were inoculated with PDX-3887. Tumors were allowed to reach 50–100 mm^3^ prior to randomization and treatment with an intraperitoneal (I.P.) injection of 100 μL vehicle [97% polyethylene glycol 300 (PEG-300, Sigma-Aldrich 202371), 1% Tween-20, and 2% DMSO], 20 mg/kg BZA (AdooQ Bioscience A11665), 50 mg/kg KCZ (Cayman Chemical 15212), or combination. Treatments were administered once a day, 5 days/week until study termination. Tumor progression was monitored using electronic caliper measurements twice weekly. Tumor volume was measured using the following equation:

$$Tumor\;volume\;\left({mm}^{3}\right)=(Width{)}^{2}\times \frac{Length}{2}$$

In the intracardiac mouse model, 6–7 week-old female athymic mice were intracardially inoculated with 1.0 × 10^5^ exponentially growing MDA-MB-231-tGLI1 breast cancer cells. Successful inoculations were confirmed using a brain bioluminescent signal within 1 h of inoculation. Mice were randomized and began receiving I.P. treatment 3 days post inoculation. Mice were treated with either vehicle (97% PEG-300, 1% Tween-20, and 2% DMSO), 10 mg/kg BZA, 50 mg/kg KCZ, or a combination. Metastatic burden was monitored throughout the study using biweekly bioluminescent imaging (BLI). Mice were administered 100 µL of 1 mg/kg d-luciferin (Perkin Elmer 122799) (Waltham, MA, USA) and imaged using the IVIS Lumina LT Series III imager (Perkin Elmer). BLI was quantified using the Living Image software version 4.7.2 (Perkin Elmer).

### 2.7. Alanine Transaminase Activity (ALT) Assay

Blood was collected through cardiac bleeds at the endpoint of all animal studies. Serum was isolated from the whole blood through centrifugation. Serum ALT activity was quantified using the ALT Assay Kit from Cayman Chemical (700260).

### 2.8. Quantitative RT-PCR

RNA was isolated using QIAGEN RNeasy (QIAGEN 74104) (Hilden, Germany). RT-qPCR was conducted as previously described [12]. Primer information is provided in Table S1.

### 2.9. Immunohistochemistry (IHC) and Tissue Microarray (TMA)

The breast cancer TMA (BR10010f) was purchased from US Biomax (Rockville, MD, USA). The paraffin-embedded breast TMA was stained for tGLI1 using a custom-made antibody previously validated in our lab (YenZym) and GP130 (Abcam ab202850) (Cambridge, United Kingdom) [14]. TGLI1 was scored based on nuclear positivity, and GP130 was scored using a histologic scoring method (H-score) as previously described [12]. IHC was conducted as previously described [14]. For IHC analysis of node-positive primary breast cancer TMA (n = 49), tumor samples were stratified based on breast cancer molecular subtype into luminal (n = 27), HER2-enriched (n = 13), and TNBC (n = 9). Samples positive for tGLI1 were labeled (tGLI1+), samples positive for GP130 were labeled (GP130+), and samples positive for both tGLI1 and GP130 were labeled (tGLI1+/GP130+). Percent positivity was calculated by dividing positive samples by the total number of samples in each group. A Chi-square test was used for statistical analyses. For IHC analysis of PDX tumors ex vivo, tumors were extracted upon endpoint. Frozen tumor sections (10 µm) were stained with either Ki-67 (CST 9027, 1:400) or cleaved PARP (CST 5625, 1:50).

### 2.10. Flow Cytometry

Adherent cells were harvested and seeded into 10 cm dishes at a seeding density of 1 × 10^6^ cells. Cells were allowed to equilibrate for 24 h at 37 °C, 5% CO_2_. For co-overexpression experiments, cells were transiently transfected using an X-tremeGENE^™^ HP DNA Transfection Reagent (Millipore Sigma 6366236001) (Burlington, MA, USA) in a reduced-serum medium for 24–48 h before harvesting for flow cytometry analysis. For treatment experiments, cells were treated with either vehicle (1% DMSO), BZA, KCZ, or combination for 24–72 h before harvesting for analyses. Collected cells were then stained with LIVE/DEAD™ Fixable Dead Cell Stain Kit (Invitrogen L23105) (Waltham, MA, USA) and either CD44 (BioLegend 103012) (San Diego, CA, USA) and CD24 (eBioscience 11024742) (San Diego, CA, USA) or the respective isotype controls (Biolegend APC Rat IgG2b 400612) (eBiosceince FITC Mouse IgG1 κ 11471482) for 20 min at room temperature. Cells were fixed on ice in 1% paraformaldehyde prior to analysis. For annexin V/PI staining (Abcam ab39758), collected cells (2.5 × 10^5^ cells/condition) were co-stained with annexin V/PI according to the manufacturer’s recommendations. The cells were incubated in the dark with antibodies at room temperature for 5 min. Flow cytometry was conducted using a BD LSRFortessa^TM^ Cell Analyzer, and analyses of samples were performed using FlowJo^TM^ V10.10.0 (BD Biosciences) (Franklin Lakes, NJ, USA) software.

### 2.11. Mammosphere Assay

For co-overexpression experiments, cells were transiently transfected using X-tremeGENE^™^ HP DNA Transfection Reagent (Millipore Sigma 6366236001) in a reduced-serum medium for 24 h. Transfected cells were harvested and seeded at a density of 1–2 × 10^3^ seeded in ultra-low attachment 24-well plates (Corning 3473). Mammosphere media was prepared as previously described [12]. Then, 100 µL of mammosphere media was administered every 48 h. Mammospheres that reached the 100 µm threshold were counted on days 3, 5, 7, and/or 10. For treatment experiments, cells were harvested and seeded at a density of 1–2 × 10^3^ seeded in ultra-low attachment 24-well plates. Treatment of either vehicle (1% DMSO), BZA, KCZ, or combination was administered 24 h after seeding. Then, 100 μL of fresh treatment media was added every 48 h until completion of the assay.

### 2.12. Aldehyde Dehydrogenase (ALDH) Activity Assay

Cells were either transfected or treated as previously described in the *Flow Cytometry* Experiments section above. Cells were harvested and prepared per the manufacturer’s instructions (Abcam ab155893). ALDH activity was measured kinetically at 405 nm every 2 min for 66 min using a microplate reader (Agilent Biotek Synergy H1) (Santa Clara, CA, USA).

## 3. Results

### 3.1. TGLI1 and IL-6R/GP130/STAT3 Pathways Are Frequently Co-Expressed and Co-Activated in HER2-Enriched Breast Cancer and TNBC

**Figure 1:**
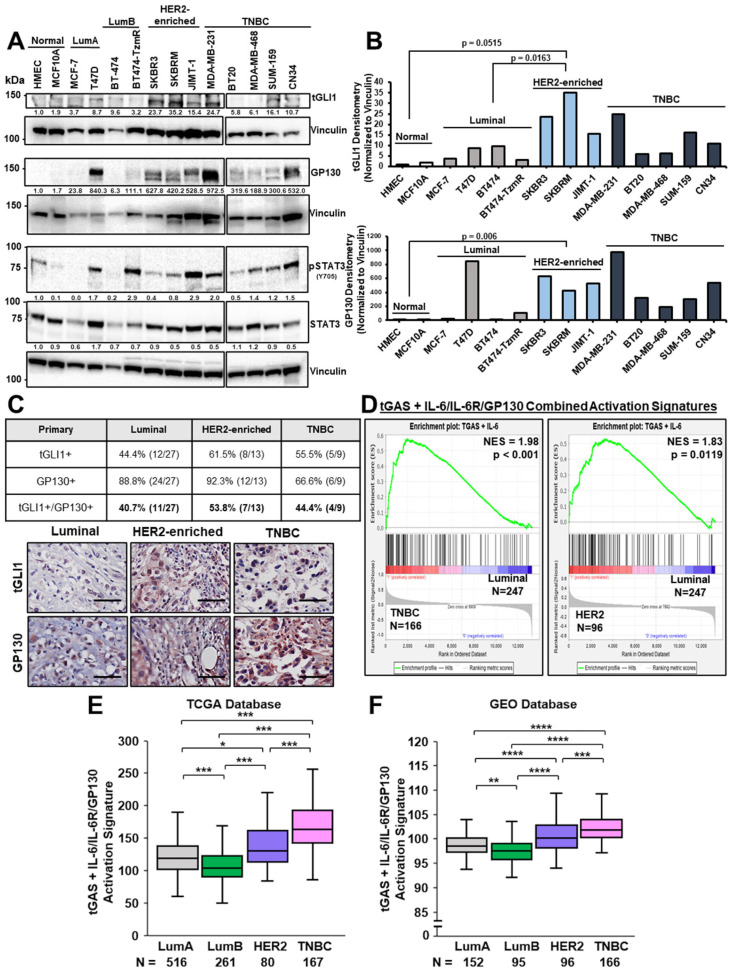
The tGLI1 and IL-6/IL-6R/GP130/STAT3 pathways are frequently co-expressed and co-activated in HER2-enriched breast cancer and TNBC. (**A**) Western blot panel of normal mammary epithelial cells and breast cancer cell lines to determine endogenous tGLI1, GP130, pSTAT3-Y705, and total STAT3 protein expression. (**B**) Western blot quantifications of tGLI1 and GP130 protein expression in breast cancer cells (normalized to Vinculin). (**C**) IHC staining of tGLI1 and GP130 expression levels in 49 node-positive primary breast tumors stratified by either luminal, HER2-enriched, or TNBC subtypes. Representative 40X images are shown (bottom). The scale bar indicates 50 μm. (**D**) GSEA analysis using GEO breast cancer datasets (GSE12276/2034/2603/5327/14020) comparing tGLI1+IL-6/IL-6R/GP130 activation signatures in TNBC versus luminal breast cancers (left) or HER2-enriched versus luminal breast cancers (right). (**E**) tGLI1+IL-6/IL-6R/GP130 activation scores across molecular subtypes of breast cancer using TCGA. (**F**) GEO was used to derive activation scores of tGLI1+IL-6/IL-6R/GP130 to analyze pathway activation across major molecular subtypes of breast cancer. Note: *, *p* < 0.05; **, *p* < 0.01; ***, *p* < 0.001; ****, *p* < 0.0001. An unpaired, two-tailed *t*-test was used in panel (**B**). One-way ANOVAs with post-hoc Tukey’s multiple comparison tests were used to compute *p*-values in panels (**E**,**F**).

We previously demonstrated that tGLI1 and STAT3 oncogenic transcription factors directly interact and functionally promote the aggressiveness of HER2-enriched breast cancer and TNBC [13]. Whether tGLI1 and GP130, an upstream regulator of STAT3, are frequently co-overexpressed and concurrently activated in breast cancer has not yet been investigated. Here, we analyzed protein expression of tGLI1, GP130, phosphorylated STAT3 (pSTAT3-Y705), and total STAT3 in normal mammary cell lines in addition to luminal A, luminal B, HER2-enriched, and TNBC breast cancer cell lines. We found tGLI1, GP130, and pSTAT3-Y705 to be frequently co-expressed in HER2-enriched breast cancer and TNBC cell lines, which are considered as the more aggressive subtypes of breast cancer (Figure 1A). Notably, there was minimal to no protein expression of tGLI1, GP130, and pSTAT3-Y705 in normal mammary epithelial cells, HMEC, and MCF10A, suggesting a therapeutic window that spares surrounding microenvironmental cells. TGLI1 and GP130 protein expressions were found to be significantly elevated in HER2-enriched breast cancer subtypes and trended for TNBC but did not reach statistical significance (Figure 1B). To further validate our findings, we analyzed a breast TMA (BR10010f) containing 49 lymph node-positive (LN+) primary breast tumor samples. IHC analysis of tGLI1 and GP130 revealed that 40.7% of luminal breast tumors co-overexpress tGLI1 and GP130 (Figure 1C). A greater frequency of tGLI1/GP130 co-overexpression, although not statistically significant, was observed in HER2-enriched and TNBC breast tumors at 53.3% and 44.4%, respectively (Figure 1C). To determine if co-expression is associated with the activation of both oncogenic signaling pathways, we conducted data mining analyses using publicly available breast cancer patient datasets. First, we created a combined activation signature (tGAS+IL-6/IL-6R/GP130) by integrating target genes known to be transcriptionally regulated by tGLI1 and IL-6/IL-6R/GP130. tGLI1 activation signature (TGAS) is comprised of genes transcriptionally regulated by tGLI1, but not GLI1, and includes VEGFA, VEGFC, VEGFR2, TEM7, HPSE, CD24, CD44, and OCT4 [9,10,11]. The IL-6/IL-6R/GP130 activation signature contains IL-6/IL-6R/GP130 and STAT3 target genes [37,38,39]. Using this combined activation signature, we found that tGLI1 and IL-6/IL-6R/GP130 signaling pathways were significantly enriched in HER2-enriched breast cancer (N = 96) and TNBC (N = 166) patients when compared to luminal subtypes (N = 247) (Figure 1D). Furthermore, tGLI1 and IL-6/IL-6R/GP130 were significantly co-activated in HER2-enriched breast cancer and TNBC when compared to luminal patients, as validated by two separate breast cancer datasets, The Cancer Genome Atlas (TCGA) (Figure 1E) and the Gene Expression Omnibus (GEO) (Figure 1F). Collectively, our results demonstrate that tGLI1 and IL-6 signaling pathways are frequently co-overexpressed, co-enriched, and co-activated in HER2-enriched breast cancers and TNBCs.

### 3.2. Co-Overexpression of tGLI1 and GP130 Enriches the Breast CSC Subpopulation in HER2-Enriched Breast Cancer and TNBC

**Figure 2:**
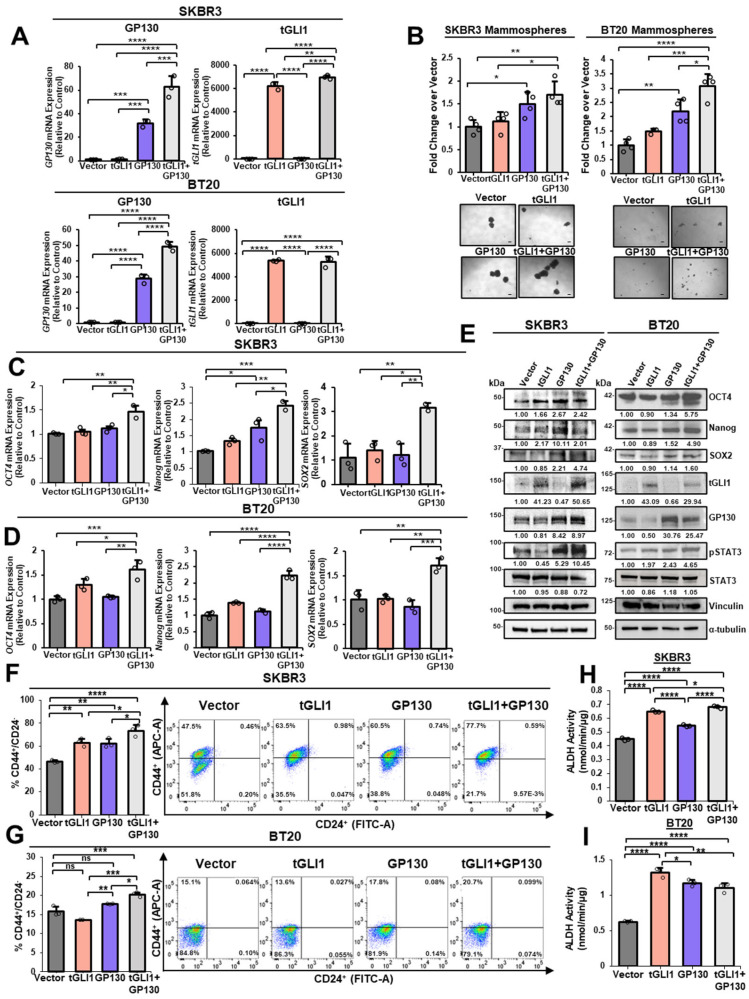
Co-overexpression of tGLI1 and GP130 enriches breast CSC phenotypes in HER2-enriched breast cancer and TNBC. (**A**) Confirmation of SKBR3 and BT20 cell lines overexpressing either vector, tGLI1, GP130, or tGLI1+GP130 using RT-qPCR analysis. (**B**) Mammosphere formation of either SKBR3 or BT20 breast cancer cells transiently transfected to overexpress vector, tGLI1, GP130, or tGLI1+GP130. The scale bar indicates 100 μm. (**C**) RT-qPCR analysis of OCT4, Nanog, and SOX2 mRNA expression in SKBR3 cells overexpressing vector, tGLI1, GP130, or tGLI1+GP130. (**D**) OCT4, Nanog, and SOX2 mRNA expression levels in BT20 cells transfected with vector, tGLI1, GP130, or tGLI1+GP130 expression vectors. (**E**) Western blot analysis to determine protein expression of OCT4, Nanog, SOX2, tGLI1, GP130, pSTAT3-Y705, and STAT3 in vector, tGLI1, GP130, or tGLI1+GP130 overexpressing SKBR3 (right) and BT20 (left) cells. Vinculin serves as a high molecular weight loading control, and α-tubulin serves as a low molecular weight loading control. (**F,G**) Quantification of CD44^+^/CD24^−^ SKBR3 (top) or BT20 (bottom) cells overexpressing vector, tGLI1, GP130, or tGLI1+GP130. Representative flow cytometry scatter plots (right). (**H,I**) Quantification of ALDH activity in both SKBR3 (top) and BT20 (bottom) cells overexpressing vector, tGLI1, GP130, or tGLI1+GP130. Note: *, *p* < 0.05; **, *p* < 0.01; ***, *p* < 0.001; ****, *p* < 0.0001. One-way ANOVAs with post-hoc Tukey’s multiple comparison test were used to calculate *p*-values.

Due to the clinical evidence that co-activation of the tGLI1 and IL-6/IL-6R/GP130 signaling pathways is associated with more aggressive subtypes in breast cancer patients (Figure 1), we next examined the role of tGLI1 and GP130 in the HER2-enriched breast cancer and TNBC stem cell subpopulation in vitro. Breast CSCs are a small subset of undifferentiated cells that play a critical role in tumorigenesis, drug resistance, and tumor recurrence [43]. First, we established an ectopic tGLI1+GP130 co-overexpression model to analyze the effects on the breast CSC subpopulation. HER2-enriched breast cancer cell line, SKBR3, and TNBC cells, BT20, were selected due to their lower endogenous expression of tGLI1, GP130, and pSTAT3-Y705 (Figure 1A). SKBR3 or BT20 cells were transiently transfected to co-overexpress either vector, tGLI1, GP130, or tGLI1+GP130. Co-overexpression of tGLI1 and GP130 was validated at the mRNA (Figure 2A) and protein expression levels (Figure 2E). Next, cells co-transfected with tGLI1+GP130 were seeded in non-adherent, mammosphere-forming conditions to enrich the breast CSC subpopulation [44]. Co-overexpression of tGLI1 and GP130 significantly increased the mammosphere-forming ability of SKBR3 and BT20 cells (Figure 2B). To elucidate how tGLI1 and GP130 may enrich breast CSCs functionally, we analyzed mRNA and protein expression of common stemness markers, including octamer-binding transcription factor 4 (OCT4), Nanog, and sex-determining region Y-box 2 (SOX2). OCT4, Nanog, and SOX2 are critical regulators of pluripotency and self-renewal and are known to be associated with poor clinical outcomes due to their role in promoting tumor-initiating cells [45]. Co-overexpression of tGLI1+GP130 upregulated mRNA expression of *OCT4*, *Nanog*, and *SOX2* when compared to control and single transfected cells in both SKBR3 (Figure 2C) and BT20 breast cancer cells (Figure 2D). Concordantly, OCT4, Nanog, and SOX2 protein expression levels were increased upon co-transfection of tGLI1+GP130 relative to the vector control in both SKBR3 and BT20 breast cancer cells (Figure 2E). Of note, pSTAT3-Y705 protein expression was increased upon GP130 and tGLI1+GP130 co-overexpression, confirming IL-6 pathway activation (Figure 2E). To further validate tGLI1 and GP130 cooperation in promoting breast CSCs, we next analyzed CD44^+^/CD24^−^ cells using flow cytometry. CD44 and CD24 are cell surface markers frequently used to identify breast CSCs, and CD44^+^/CD24^−^ cells are considered stem cell-like [46]. In agreement with our findings, tGLI1 and GP130 co-overexpression significantly increased the CD44^+^/CD24^−^ breast cancer cell population in both SKBR3 (Figure 2F) and BT20 cells (Figure 2G). Moreover, aldehyde dehydrogenase (ALDH) activity, which is enriched in the stem cell subpopulation [47], was significantly elevated in SKBR3 (Figure 2H) upon co-overexpression of tGLI1+GP130 relative to vector or single transfection conditions. ALDH activity was increased by tGLI1, GP130, and tGLI1+GP130 overexpression conditions when compared to vector in BT20 cells (Figure 2I). Our results collectively demonstrate that tGLI1 and GP130 co-overexpression promote breast CSC phenotypes in HER2-enriched and TNBCs, thereby underscoring their clinical utility to be dual-targeted as a novel combinatorial therapy for breast cancer.

### 3.3. The Combination of KCZ and BZA Is Synergistic Against HER2-Enriched Breast Cancer and TNBC CSCs

**Figure 3:**
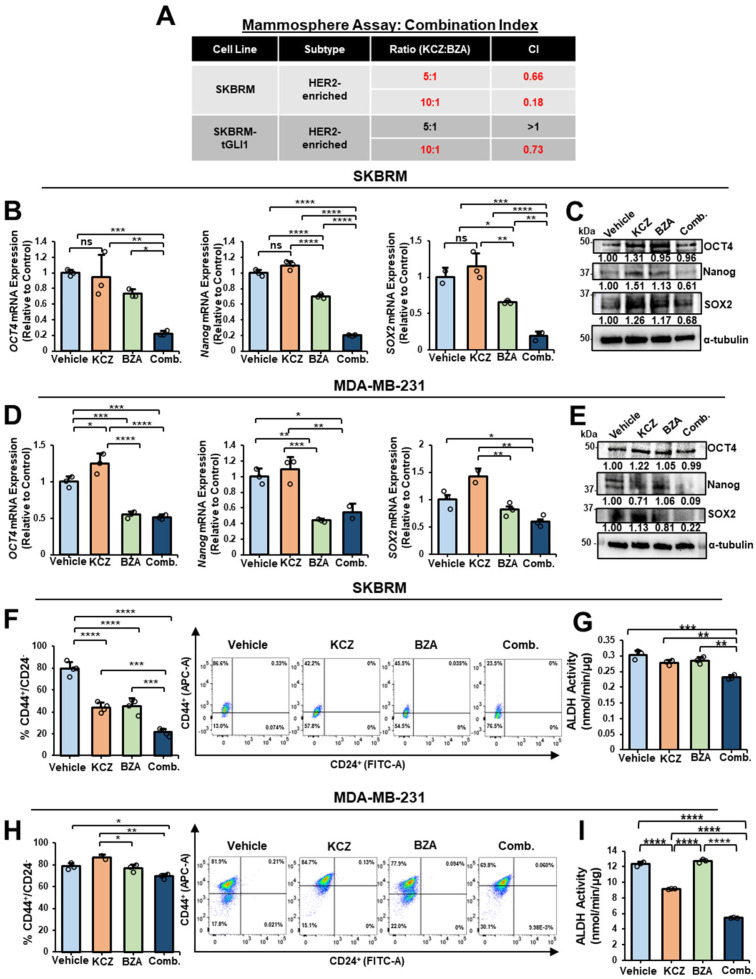
The combination of KCZ and BZA is synergistic against HER2-enriched breast cancer and TNBC CSCs. (**A**) CIs of SKBRM and SKBRM-tGLI1 mammospheres treated with either 5:1 or 10:1 ratio of KCZ+BZA. CIs were derived using the CompuSyn software V1.0 based on the Chou–Talalay theorem to determine the effects between two compounds: additive (CI = 1), synergistic (CI < 1), or antagonistic (CI > 1). (**B**) RT-qPCR analysis of *OCT4, Nanog*, and *SOX2* mRNA expression levels in SKBRM breast cancer cells treated with either vehicle, KCZ, BZA, or combination at a 5:1 ratio (KCZ+BZA). (**C**) Protein expression of OCT4, Nanog, and SOX2 after vehicle, KCZ, BZA, or 5:1 combination treatment in SKBRM breast cancer cells (normalized to α-tubulin). (**D**) Effect of vehicle, KCZ, BZA, or 5:1 KCZ+BZA treatment on *OCT4, Nanog*, and *SOX2* mRNA expression levels in MDA-MB-231 breast cancer cells using RT-qPCR. (**E**) Western blot analysis of OCT4, Nanog, and SOX2 protein expression post-treatment in MDA-MB-231 cells (normalized to α-tubulin). (**F**) Quantification of CD44^+^/CD24^−^ after 5:1 (KCZ+BZA) combination treatment in SKBRM cells. Representative flow cytometry graphs (right). (**G**) ALDH activity (nmol/min/µg) of SKBRM cells after treatment with vehicle, KCZ, BZA, or combination. (**H**) Representative scatter plots (right) and flow cytometry analysis of CD44^+^/CD24^−^ MDA-MB-231 cells after KCZ+BZA treatment. (**I**) Quantification of ALDH activity after 5:1 KCZ+BZA combination treatment in MDA-MB-231 cells. Note: *, *p* < 0.05; **, *p* < 0.01; ***, *p* < 0.001; ****, *p* < 0.0001; one-way ANOVAs with post-hoc Tukey’s multiple comparison tests were used to calculate *p*-values for panels (**B**,**D**,**F**–**I**).

Since breast CSCs mediate tumor relapse and tGLI1+GP130 co-overexpression enriches breast CSCs, we next investigated if concurrent inhibition of tGLI1 and GP130 could be an effective novel combinatorial therapy to suppress breast CSCs. To alleviate unwanted toxicity and expedite the drug approval pipeline, we repurposed FDA-approved compounds for our combination therapy. We previously reported an FDA-approved antifungal, KCZ, to be the first compound to selectively target tGLI1, but not wild-type GLI1 [12]. Multiple FDA-approved compounds have shown GP130 inhibitory effects; however, GP130 is ubiquitously expressed and is the primary membrane-bound signaling receptor for the IL-6 family of cytokines [48]. We proceeded to evaluate FDA-approved BZA due to its selective effects against downstream IL-6 signaling without impacting signaling mediated by other cytokines [31]. BZA is also reported to be efficacious against TNBC growth in vitro and in vivo [32]. We first used mammosphere formation assays to determine whether KCZ and BZA could synergize against breast CSCs. HER2-enriched breast cancer cells, SKBRM, and TNBC cells, MDA-MB-231, were selected due to high co-expression of tGLI1 and GP130 (Figure 1A). Since GP130 is ubiquitously expressed, breast cancer cells stably overexpressing tGLI1 were also used to evaluate KCZ+BZA synergy against breast CSCs with comparable levels of tGLI1 and GP130. SKBRM and MDA-MB-231 cells stably overexpressing tGLI1 (SKBRM-tGLI1 and MDA-MB-231-tGLI1, respectively) were previously established [14]. MDA-MB-231 cells do not form spheres under our mammosphere-forming conditions and were, therefore, later evaluated in monolayer conditions. To determine the effects between KCZ and BZA against SKBRM mammospheres, a Combination Index (CI) was derived using the Chou-Talalay method, which defines drug combinations as either additive (CI = 1), synergistic (CI < 1), or antagonistic (CI > 1) [40]. The ratios (5:1 or 10:1 KCZ+BZA) were selected based on individual compound IC50s. In summary, a 5:1 and 10:1 ratio of KCZ+BZA was synergistic against SKBRM mammospheres (Figure 3A). A 10:1 ratio of KCZ+BZA synergized against SKBRM cells stably overexpressing tGLI1 (Figure 3A). Furthermore, a 5:1 ratio of KCZ+BZA reduced the mRNA expression of stemness markers *OCT4*, *Nanog*, and *SOX2* when compared to vehicle and monotherapies in SKBRM cells (Figure 3B), yet only the protein expression of Nanog and SOX2 was reduced (Figure 3C). Similar results were obtained using the TNBC cell line, MDA-MB-231 (Figure 3D,E). Despite the reduction in *OCT4* mRNA transcripts, combination treatment yielded little to no reduction in OCT4 protein expression levels, indicating potential activation by alternative pathways such as Wnt, PI3K, and HIF2-α [49]. To further analyze KCZ+BZA combination therapy against the breast CSC subpopulation, SKBRM, and MDA-MB-231 cells were treated with either vehicle, KCZ, BZA, or a combination of KCZ+BZA to evaluate changes in CD44^+^/CD24^−^ cells using flow cytometry. KCZ+BZA combination treatment significantly reduced CD44^+^/CD24^−^ SKBRM cells when compared to vehicles or monotherapies (Figure 3F). A significant reduction was observed in MDA-MB-231 cells; however, combination treatment was not more effective than BZA alone (Figure 3H). Combination treatment also suppressed CD44^+^/CD24^−^ cells compared to vehicle and single treatments in MDA-MB-468 TNBC cells (Figure S1). Upon evaluation of ALDH activity, combination treatment significantly reduced ALDH activity in both SKBRM cells (Figure 3G) and MDA-MB-231 cells (Figure 3I) when compared to vehicle or single agents. Together, these results demonstrate that simultaneous inhibition of tGLI1 and GP130 using KCZ and BZA, respectively, is an effective treatment strategy to suppress CSC phenotypes in HER2-enriched breast cancer and TNBC.

### 3.4. Co-Treatment with KCZ+BZA Reduces HER2-Enriched Breast Cancer and TNBC Cell Viability and Migratory Ability

**Figure 4:**
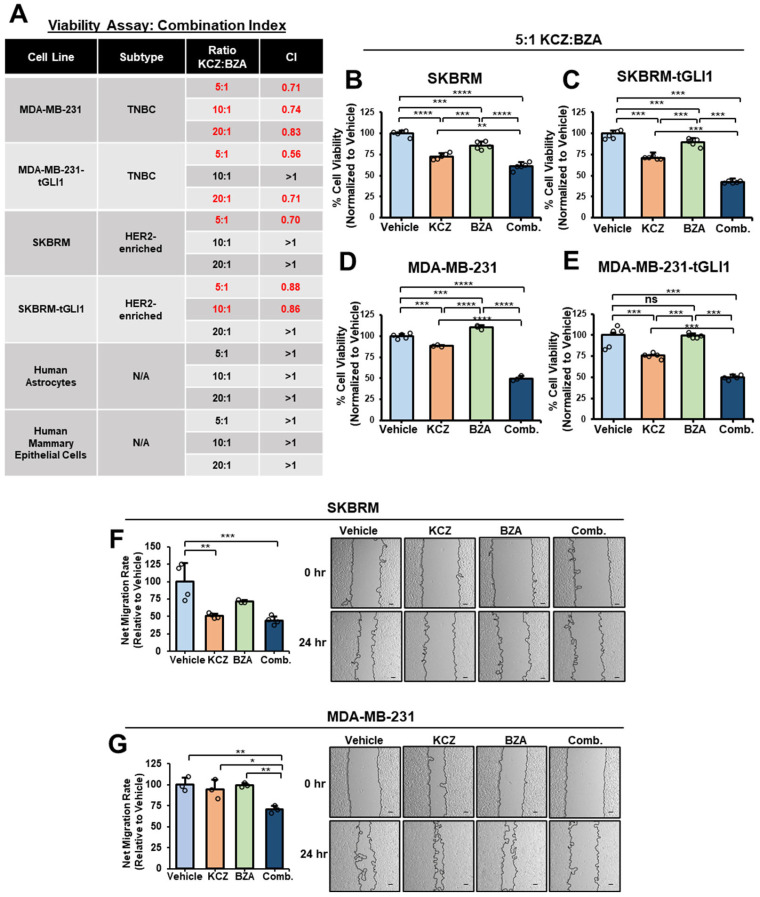
Co-treatment with KCZ+BZA reduces HER2-enriched breast cancer and TNBC cell viability and migratory ability. (**A**) KCZ and BZA exhibit synergism (CI < 1) in SKBRM, SKBRM-tGLI1, MDA-MB-231, and MDA-MB-231-tGLI1 breast cancer cells using cell-based viability assays. KCZ+BZA treatment on normal cells (bottom). Cells were seeded in 96-well plates and treated with 5:1, 10:1, or 20:1 ratio of KCZ+BZA for 48 h. CIs were derived using CompuSyn software. (**B–E**) Representative 5:1 cell viability graphs. (**F**) Net migration rate of SKBRM cells after 24-h treatment of vehicle, KCZ, BZA, or combination. The scale bar indicates 100 μm. (**G**) Scratch-wound assay of MDA-MB-231 cells treated for 24 h with vehicle, KCZ, BZA, or combination. Note: *, *p* < 0.05; **, *p* < 0.01; ***, *p* < 0.001; ****, *p* < 0.0001; one-way ANOVAs with post-hoc Tukey’s multiple comparison tests were used to calculate *p*-values for panels (**B**–**G**).

To further analyze KCZ+BZA combinatorial treatment, cell-based viability assays were conducted to determine if KCZ+BZA combination therapy synergizes against HER2-enriched breast cancer and TNBC cells under adherent conditions. Multiple doses within a 5:1, 10:1, or 20:1 ratio of KCZ+BZA were screened to derive a CI. KCZ synergized with BZA at a 5:1 ratio in SKBRM cells (Figure 4A,B). The KCZ+BZA combination also displayed synergy in the TNBC cell line, MDA-MB-231, at all ratios (Figure 4A,D and Figure S2C). KCZ also synergized with BZA in tGLI1-overexpressing cells (Figure 4A,C,E and Figure S2B,D), showing the greatest synergy in MDA-MB-231-tGLI1 breast cancer cells (Figure 4A). Since the KCZ and BZA combination was synergistic at a 5:1 ratio throughout all cell lines tested, we selected the 5:1 ratio for evaluation in all in vitro assays (Figure 4A). To examine potential toxicity to surrounding microenvironmental cells, we tested KCZ and BZA combination therapy on normal mammary epithelial cells as well as the most abundant cell type in the brain, human astrocytes, since both drugs are reported to cross the blood-brain barrier (BBB) [50,51]. Notably, KCZ+BZA yielded no synergism at any ratio evaluated in MCF10A and human astrocytes (Figure 4A, bottom). Metastasis is a multi-step process including local migration, invasion, and eventually colonization in a distant organ. Since co-treatment of KCZ+BZA suppressed breast CSCs and breast cancer cell viability, we next examined whether this combination treatment has an effect on breast cancer cell migration, which is critical for early-stage metastasis. SKBRM or MDA-MB-231 cells were subjected to a scratch-wound assay and monitored for migration post treatment. A cell-based viability assay was conducted in tandem (Figure S3) to derive a proliferation-independent net migratory rate (Figure 4F,G). Combination treatment reduced the migratory ability of SKBRM (Figure 4F) and MDA-MB-231 cells (Figure 4G) when compared to vehicle treatment. Since KCZ+BZA combination treatment targets breast cancer cells, spares normal cells, and reduces migration, these data further support the clinical utility of dual-targeting both tGLI1 and GP130 for HER2-enriched breast cancer and TNBC.

### 3.5. Dual-Inhibition Using KCZ+BZA Induces Apoptosis in an AR-Independent Manner and Downregulates tGLI1+STAT3 Transcriptional Targets Cep70, UPF3A, and RRas2

**Figure 5:**
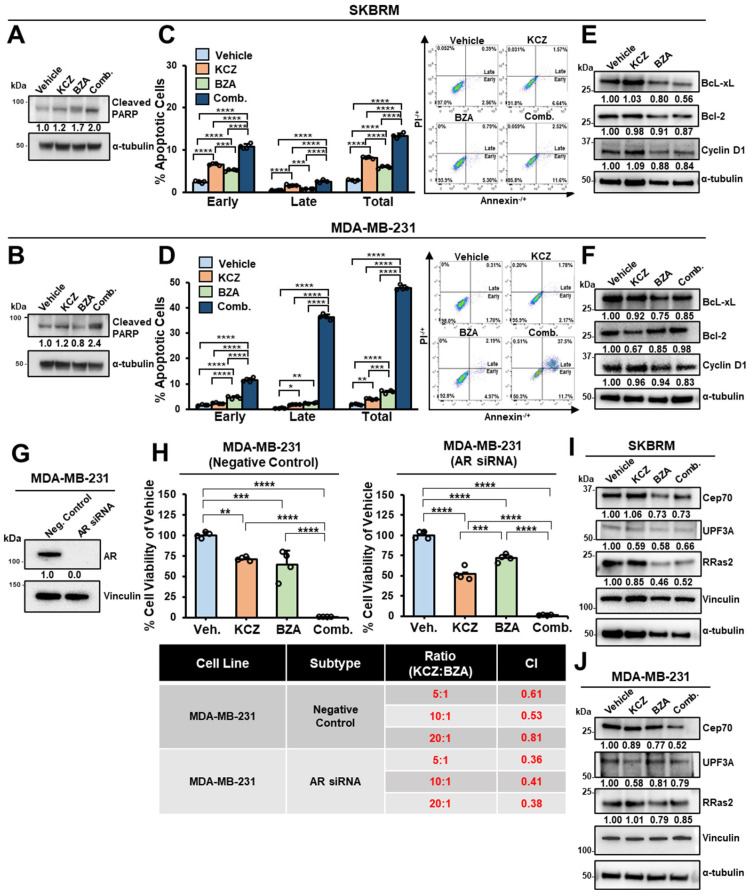
Dual inhibition using KCZ+BZA induces apoptosis, is AR-independent, and downregulates tGLI1+STAT3 co-targets Cep70, UPF3A, and RRas2. (**A,B**) Western blot analysis of cleaved PARP in SKBRM cells and MDA-MB-231 cells. (**C**) Annexin V/PI flow cytometry co-staining of SKBRM cells treated with vehicle, KCZ, BZA, and combination. (**D**) KCZ+BZA combination treatment induces apoptosis in MDA-MB-231 cells using flow cytometry analysis of Annexin V/PI co-staining. (**E,F**) Protein analysis of anti-apoptotic markers (BcL-xL and Bcl-2) and cell cycle regulators (cyclin D1) in SKBRM and MDA-MB-231 breast cancer cells. (**G**) MDA-MB-231 breast cancer cells were transiently transfected with either negative control (scrambled siRNA) or AR siRNA. Confirmation of AR knockdown via western blot analysis. (**H**) Negative control or AR knockdown MDA-MB-231 cells were treated with vehicle, KCZ, BZA, or combination for 48 h. Representative cell viability graphs (top). CIs are shown (bottom). (**I,J**) Western blot analysis of tGLI1-STAT3 targets Cep70, UPF3A, and RRas2 in SKBRM and MDA-MB-231 cells after treatment of vehicle, KCZ, BZA, or combination. Note: *, *p* < 0.05; **, *p* < 0.01; ***, *p* < 0.001; ****, *p* < 0.0001; one-way ANOVAs with post-hoc Tukey’s multiple comparison tests were used to calculate *p*-values for panels (**C**,**D**,**H**).

Due to the stark reduction in breast cancer cell viability after combination treatment (Figure 4B–E), we next asked if combination efficacy is mediated through the induction of apoptosis. To test this, we analyzed cleaved poly-ADP ribose polymerase (PARP) protein expression levels after treatment of either vehicle, KCZ, BZA, or combination. Full-length PARP is a DNA damage sensor that is cleaved into fragments (cleaved PARP) under caspase-dependent apoptosis [52]. Combination treatment induced cleaved PARP protein expression when compared to vehicles or monotherapies in both SKBRM (Figure 5A) and MDA-MB-231 cells (Figure 5B). As an additional marker for cell apoptosis, annexin V and propidium iodide (PI) co-staining was conducted using flow cytometry analysis. Fluorescently labeled Annexin V binds phosphatidylserines, which, during active apoptosis, become exposed on the outside of the plasma membranes [53]. PI stains DNA and is used to differentiate apoptotic cells from necrotic cells. Co-staining with annexin V and PI can distinguish cells undergoing early apoptosis (Annexin V^+^/PI^−^), late apoptosis (Annexin V^+^/PI^+^), and necrosis (Annexin V^−^/PI^+^). Combination treatment increased early, late, and total apoptosis in SKBRM (Figure 5C) and MDA-MB-231 cells (Figure 5D). To complement our findings, we evaluated the protein expression levels of two anti-apoptotic markers, BcL-xL and Bcl-2 [54]. It is worth mentioning that BcL-xL is reported to be a more functional anti-apoptotic protein than Bcl-2 in breast cancer cells [55]. We found that co-treatment of KCZ+BZA decreased BcL-xL and Bcl-2 protein levels in SKBRM cells (Figure 5E). Particularly, only BcL-xL was reduced upon combination treatment in MDA-MB-231 cells (Figure 5F). Moreover, STAT3 upregulates cyclin D1 to regulate cell cycle progression in breast cancer [56]. Co-inhibition reduced protein expression of cyclin D1 in both SKBRM (Figure 5E) and MDA-MB-231 (Figure 5F) cells, further reinforcing the ability of the combination treatment to suppress cellular growth through induction of apoptosis. To address the mechanisms underlying KCZ+BZA combination treatment, we first examined additional known KCZ molecular targets. In addition to GP130, BZA targets the ER to function as a SERM. In the current study, we focus on ER-negative breast cancer subtypes and, therefore, is not a viable mechanism of action for our combinatorial regimen. KCZ is a potent inhibitor of cytochrome P450 3A4 (CYP3A4) and can function as an androgen receptor (AR) antagonist [57,58]. To determine if KCZ functions through CYP3A4 or AR in our combination treatment studies, we evaluated target availability in normal and breast cancer cell lines (Figure S4A). Notably, CYP3A4 is not expressed in the cell lines frequently used in our in vitro models, namely SKBRM or MDA-MB-231. AR is expressed in MDA-MB-231 breast cancer cells and could be a potential mechanism of action (Figure S4A). To address KCZ dependency on AR in our models, we knocked down AR in MDA-MB-231 breast cancer cells using siRNA (Figure 5G). Three siRNAs were validated at 24 and 48 h to confirm extended knockdown (Figure S4B). MDA-MB-231 cells transfected with negative control or AR siRNA were seeded in cell-based viability assays and treated with vehicle, KCZ, BZA, or combination. KCZ+BZA co-treatment was synergistic at all ratios irrespective of AR expression, demonstrating that KCZ is not dependent on AR in our models (Figure 5H). It is known that KCZ disrupts tGLI1 transcriptional activity and that BZA directly binds GP130 [12,32], but whether KCZ+BZA combination treatment suppresses tGLI1-STAT3′s ability to co-regulate their transcriptional targets is unknown. TGLI1 and STAT3 transcription factors interact to co-regulate target genes, Cep70, UPF3A, and RRas2, to mediate aggressive phenotypes in breast cancer [13]. KCZ+BZA co-treatment reduced protein expression of Cep70, UPF3A, and RRas2 (Figure 5I,J), shedding new light on the underlying mechanism of combination treatment in HER2-enriched breast cancer and TNBC.

### 3.6. KCZ+BZA Combination Treatment Suppresses Tumor Growth, Proliferation, and Induces Apoptosis in TNBC PDX Mouse Model

**Figure 6:**
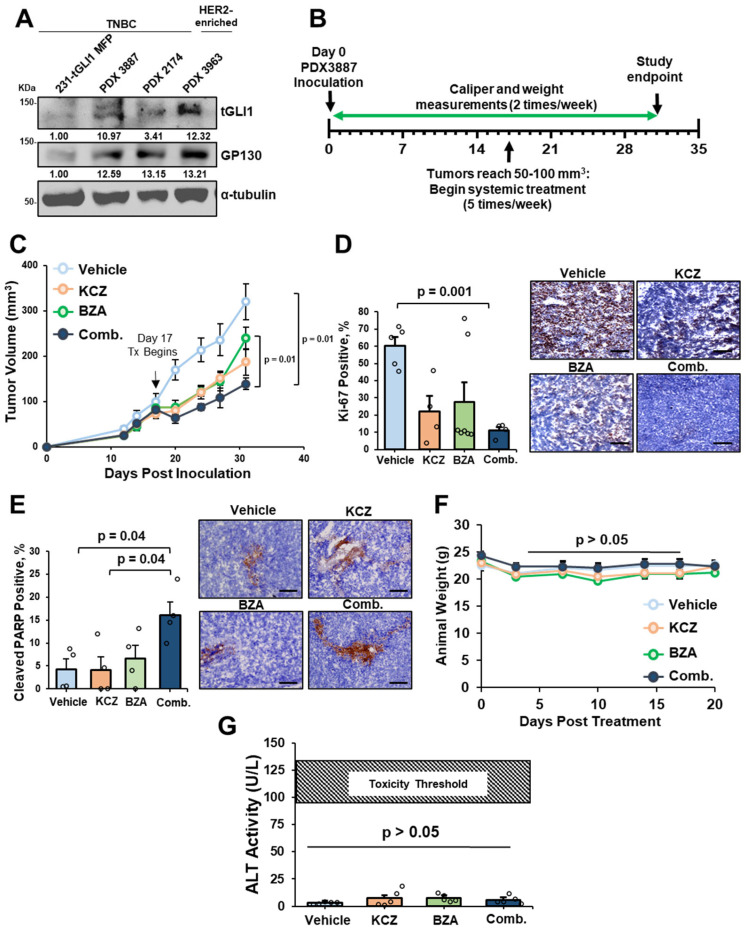
KCZ+BZA combination treatment suppresses tumor growth, and proliferation, and induces apoptosis in the TNBC PDX mouse model. (**A**) Western blot analysis of PDX lines to assess target protein expression of tGLI1 and GP130 (normalized to α-tubulin). MDA-MB-231-tGLI1 MFP is provided as a positive control. (**B**) Schematic of PDX-3887 MFP mouse model. 4–5 week-old athymic mice were inoculated with PDX-3887. Tumors reached 50–100 mm^3^, and mice were randomized prior to I.P. treatment of vehicle, KCZ (50 mg/kg), BZA (20 mg/kg), or combination (N = 7–9 mice/group). Caliper and weight measurements were recorded twice weekly. Tumors were harvested upon endpoint. (**C**) Treatment effects on tumor growth over time. (**D**) IHC analysis of PDX-3887 tumors for Ki-67 positive cells post treatment. Representative 20× images (right). The scale bar indicates 100 μm. (**E**) IHC analysis of tumors treated with vehicle, KCZ, BZA, or combination. Quantification for nuclear positivity of cleaved PARP (left), representative 20× images (right), and scale bar indicates 100 μm. (**F**) Average mouse weights over the course of the study. (**G**) Circulating ALT levels between treatment groups. Data are presented as mean ± SEM. One-way ANOVA with Tukey’s multiple comparison post-hoc test was used to compute *p*-values for panels (**D**,**E**,**G**). Two-way ANOVAs were used to calculate *p*-values with a mixed-effects analysis for panels (**C**,**F**).

Since KCZ+BZA combination treatment synergistically suppresses viability and induces apoptosis in HER2-enriched breast cancer and TNBC cells in vitro, we next investigated if KCZ+BZA combination treatment could inhibit breast tumor progression. To provide a translational model, we used a TNBC PDX-3887 due to its high endogenous protein expression of both tGLI1 and GP130 (Figure 6A). We proceeded with the TNBC subtype due to a greater increase in tGAS+IL-6/IL-6R/GP130 co-activation in TNBC patients when compared to the HER2-enriched breast cancer subtype (Figure 1E,F). PDX-3887 was implanted in the mammary fat pad (MFP) of female athymic mice, and tumors reached 50–100 mm^3^ before we initiated systemic intraperitoneal (I.P.) treatment of either vehicle, KCZ (50 mg/kg), BZA (20 mg/kg), or combination (Figure 6B). Electronic caliper measurements were taken twice weekly to monitor tumor growth throughout the study. KCZ+BZA combination treatment significantly reduced TNBC tumor growth over time when compared to both vehicle and BZA treatment (Figure 6C). Although trending, combination treatment was not more effective than KCZ alone (Figure 6C). Ex vivo IHC analysis of the breast cancer tumors revealed that KCZ+BZA combination therapy significantly reduced tumoral proliferation, as measured by Ki-67 when compared to vehicle-treated mice (Figure 6D). Combinatorial treatment also induced apoptosis, as indicated by cleaved PARP positivity, when compared to vehicle- or KCZ-treated mice (Figure 6E). Despite the FDA approval of KCZ and BZA as single agents, their novel combination warranted investigation of treatment-induced toxicity. To test this, we monitored animal weights throughout the study as an indicator of acute toxicity. There was no significant deviation in average animal weights in any treatment group, indicating no acute toxicity between treatments (Figure 6F). KCZ is a known substrate and inhibitor of CYP3A4, which, in turn, can interfere with the clearance rate of other compounds co-administered with KCZ [59]. Previous clinical studies have investigated KCZ combined with Taxols to increase chemotherapeutic concentrations for the treatment of cancer [60,61]. Of note, BZA is primarily metabolized through glucuronidation, and, therefore, little to no CYP-mediated metabolism is used for clearance [62]. To address potential acute liver toxicity, we analyzed alanine transaminase (ALT) activity levels in mouse sera ex vivo. ALT is found primarily in the liver, and lower levels can be detected in the heart and muscle cells. Upon disease states, such as drug-induced liver toxicity, ALT can be released into the serum and is frequently used as a biomarker for acute liver toxicity [63]. We found no significant difference in serum ALT activity between treatment groups, and no groups reached the previously reported toxicity threshold values (109 ± 18 U/L) using thioacetamide (Figure 6G) [64]. Herein, we demonstrate that KCZ+BZA combinatorial treatment suppresses TNBC PDX tumor progression through inhibition of proliferation and induction of apoptosis with minimal to no drug toxicity.

### 3.7. KCZ+BZA Combination Reduces Multi-Organ Metastasis, Increases Overall Survival, and Metastasis-Free Survival (MFS) Using a TNBC Intracardiac Mouse Model

**Figure 7:**
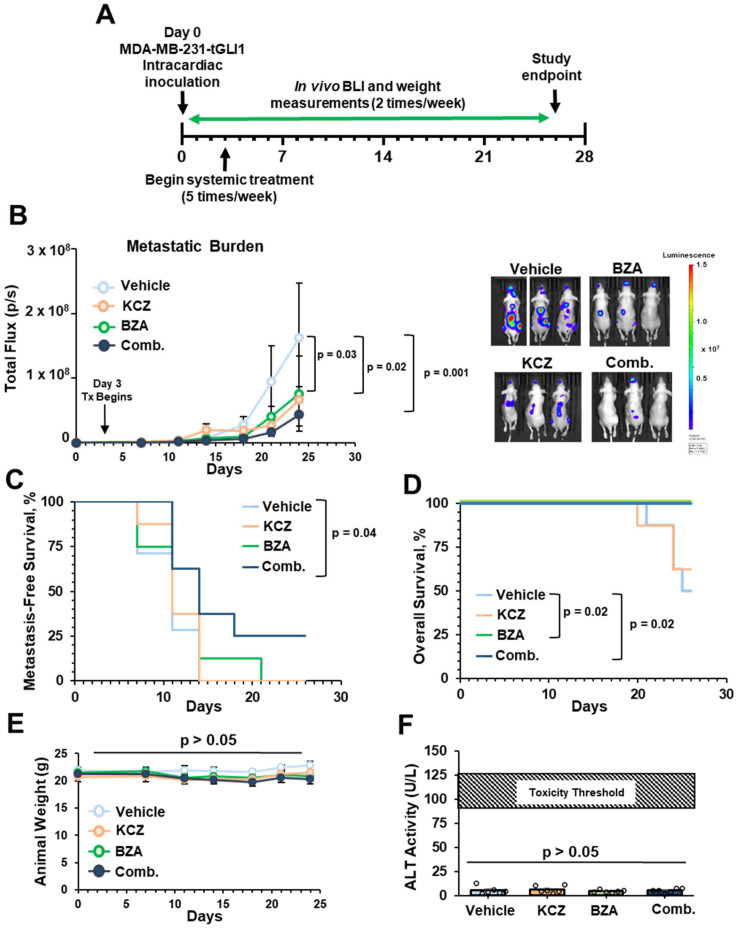
KCZ+BZA combination reduces multi-organ metastasis, increases overall survival, and MFS using a TNBC intracardiac mouse model. (**A**) Schematic of intracardiac mouse model. 6–7 week-old athymic nude mice were inoculated with luciferase-expressing MDA-MB-231-tGLI1 cells intracardially via the left ventricle. Successful inoculations were randomized and treatment of vehicle (N = 7), KCZ (50 mg/kg; N = 8), BZA (10 mg/kg; N = 8), or combination (comb; N = 8) was administered 3 days later via I.P. injection. Mice were imaged two times/week to monitor the formation and growth of metastases. N = 7–8 mice/group. (**B**) Weekly average of mice metastatic burden throughout the study using BLI. Representative images (right). (**C**) MFS between treatment groups to depict metastatic incidence. The median in days: vehicle = 11, KCZ = 11, BZA = 11, Comb. = 14). (**D**) Overall survival between vehicle, KCZ, BZA, and combination treated mice. (Median in days: vehicle = 26, KCZ = not reached, BZA= not reached, Comb. = not reached). (**E**) Average animal weight throughout the course of the study. (**F**) Serum ALT levels between vehicle, KCZ, BZA, and combination-treated mice upon the endpoint of the study. Data are represented as mean ± SEM. One-way ANOVAs with Tukey’s multiple comparison post-hoc test were used to compute *p*-values for panel (**F**). Two-way ANOVAs were used to calculate *p*-values with a mixed-effects analysis for panels (**B**,**E**). Kaplan-Meier and log-rank tests were used to compute *p*-values in panels (**C**,**D**).

Despite improvements in detection methods and early-stage diagnosis, distant metastases account for the majority of breast-cancer-related deaths [65]. Upon development of distant metastases, the 5-year survival rate for breast cancer patients plummets from 99% to a dismal 30% [1]. Risk factors include, but are not limited to, the stage upon diagnosis, tumor size, and importantly, molecular subtype. HER2-enriched breast cancer and TNBC subtypes maintain the highest propensity to metastasize, and since TNBCs are difficult to treat, we next investigated whether dual-targeting of tGLI1 and GP130 can inhibit TNBC metastasis. Using an intracardiac mouse model, we can model several steps involved in the metastasis of circulating tumor cells, including evasion of anoikis, extravasation, and colonization of breast cancer cells to a distant site. Due to the efficacy of KCZ+BZA combination therapy against MDA-MB-231-tGLI1 breast cancer cells, as indicated by the lowest CI (Figure 4A), we proceeded to use the TNBC MDA-MB-231-tGLI1 overexpressing cells for our intracardiac mouse model. Luciferase-expressing MDA-MB-231-tGLI1 cells were intracardially inoculated into the left ventricle of female athymic nude mice. The success of inoculations was monitored by bioluminescence imaging (BLI) within 1 h of inoculation. Successfully inoculated mice were then randomized 3 days post-inoculation and treated with either vehicle, KCZ (50 mg/kg), BZA (10 mg/kg), or combination via I.P. injection (Figure 7A). While mice treated with either KCZ or BZA monotherapies had a significant reduction in metastatic burden when compared to the vehicle, mice that received KCZ+BZA combination therapy displayed enhanced suppression of metastatic burden (Figure 7B). Combination treatment significantly prolonged metastasis-free survival (MFS) when compared to vehicle-treated mice (Figure 7C). Moreover, both BZA and KCZ+BZA combination treatments significantly prolonged the overall survival when compared to vehicle-treated mice (Figure 7D). All treatments were well tolerated as indicated by no significant difference between average animal weights throughout the study (Figure 7E). There was no acute liver toxicity induced between treatments, as determined by the ALT activity assay (Figure 7F). Taken together, our results demonstrate, for the first time, that the dual inhibition of tGLI1 and GP130 reduces TNBC metastasis without inducing significant toxicity.

### 3.8. TGLI1 and IL-6/IL-6R/GP130 Co-Activation Is Associated with Worse MFS in TNBC

**Figure 8:**
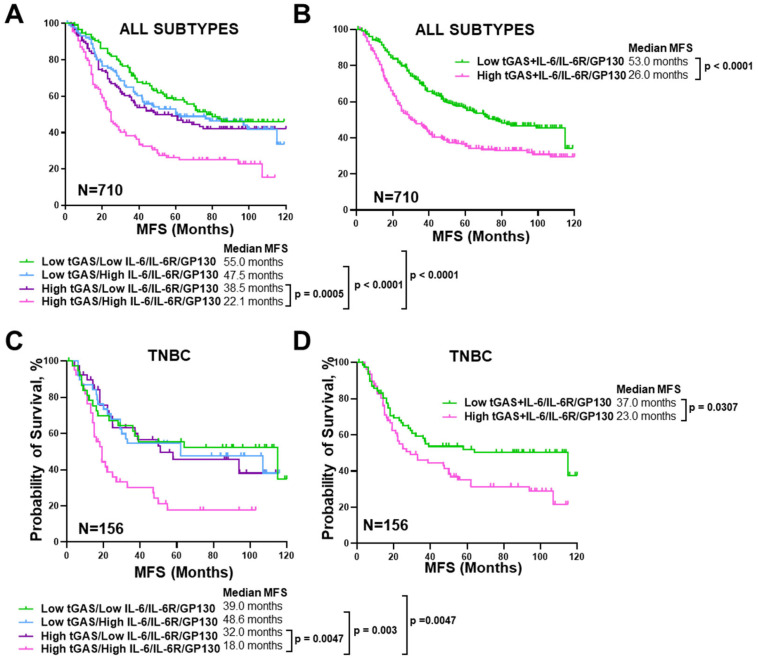
TGLI1 and IL-6/IL-6R/GP130 co-activation is associated with worse MFS in TNBC. (**A**) Using the Kaplan-Meier analysis, log-rank analyses, GEO datasets, and the tGLI1 and IL-6/IL-6R/GP130 activation signatures, 710 breast cancer patients were stratified into four groups based on the extent of pathway activation for MFS. Median MFS times are indicated in months. (**B**) MFS of breast cancer patients with either a high or low combined tGAS+IL-6/IL-6R/GP130 activation scores. (**C**) Kaplan-Meier analysis of MFS in 156 TNBC patients stratified into four groups based on the extent of tGAS or IL-6/IL-6R/GP130 pathway activation. (**D**) High versus low combined tGAS+IL-6/IL-6R/GP130 activation scores were analyzed for TNBC MFS (N = 156).

Since dual-targeting tGLI1 and GP130 suppresses metastatic burden and prolongs MFS in vivo (Figure 7B,C), we next wanted to examine the clinical relevance of tGLI1 and IL-6/IL-6R/GP130 in breast cancer metastasis using publicly available patient datasets. Using the provided MFS information in the curated GEO breast cancer patient dataset used in (Figure 1F), breast cancer patients were stratified by high versus low tGAS and IL-6/IL-6R/GP130 activation scores. Breast cancer patients with low tGLI1 and IL-6 pathway activity, as indicated by low tGAS and low IL-6/IL-6R/GP130 activation scores, had a prolonged MFS at 55.0 months (Figure 8A). Conversely, breast cancer patients with high tGAS and high IL-6/IL-6R/GP130 activation scores had a significantly shortened MFS at a dismal 22.1 months (Figure 8A). Furthermore, breast cancer patients with a combined high tGAS+IL-6/IL-6R/GP130 activation had worse MFS when compared to patients with low tGAS+IL-6/IL-6R/GP130 activation (Figure 8B). We further stratified our data based on breast cancer subtype information (Figure 8 and Figure S5). High tGAS+IL-6/IL-6R/GP130 activation did not significantly impact MFS in luminal breast cancer patients when compared to low tGAS+IL-6/IL-6R/GP130 (Figure S5B). Conversely, TNBC patients with high tGAS and high IL-6/IL-6R/GP130 had shortened MFS when compared to a low of either or both tGAS and IL-6/IL-6R/GP130, rendering both pathways therapeutically relevant for TNBC metastasis (Figure 8C). TNBC patients with a combined high tGAS+IL-6/IL-6R/GP130 had worse MFS (23 months) relative to patients with low tGAS+IL-6/IL-6R/GP130 (37 months) (Figure 8D). Collectively, our results demonstrate that tGLI1 and IL-6/IL-6R/GP130 signaling pathways are frequently co-overexpressed and co-activated and that co-activation is associated with worse MFS in HER2-enriched breast cancer and TNBC.

## 4. Discussion

In this study, we made the following novel observations: (1) tGLI1, GP130, and pSTAT3 are frequently co-expressed in HER2-enriched and triple-negative breast carcinomas; (2) tGLI1+IL-6/IL-6R/GP130 signaling is co-enriched and co-activated in HER2-enriched breast cancers and TNBCs; (3) tGLI1+IL-6/IL-6R/GP130 is associated with breast cancer metastasis; (4) tGLI1 cooperates with GP130 to promote the CSC subpopulation through the upregulation of stemness markers OCT4, Nanog, and SOX2; (5) Through these impactful observations, our study uncovers the novel co-activation of tGLI1 and GP130 and highlights their prognostic value as co-targets for HER2-enriched breast cancer and TNBC metastasis; (6) Our study also identifies a novel synergistic combinatorial treatment that operates through the dual inhibition of tGLI1 and GP130 by repurposing FDA-approved small-molecule compounds, KCZ and BZA. This approach leads to the downregulation of stemness markers Nanog and SOX2, suppression of breast CSCs, and the consequent reduction of metastatic burden and increase in overall survival using a TNBC intracardiac mouse model.

Independently, tGLI1 and GP130 have been previously investigated as viable therapeutic targets in breast cancer. We previously discovered the first tGLI1 inhibitors, KCZ and its novel derivative KCZ-7, to directly suppress tGLI1 transcriptional activity and inhibit tGLI1-mediated breast cancer brain metastasis (BCBM) in vivo [12]. To further assess KCZ BBB penetrance and alteration of tGLI1 signaling in clinical samples, our lab initiated a Phase 0 window-of-opportunity clinical trial to study the effects of KCZ in BCBM and recurrent glioma patients (NCT03796273). Currently, KCZ and KCZ-7 are the only identified tGLI1-specific inhibitors reported, thereby warranting further investigation into the development of additional KCZ derivatives to target tGLI1. Notably, KCZ is a known CYP3A4 inhibitor and should be closely evaluated when used in combinatorial regimens, specifically with CYP3A4 substrates. If combined with a CYP3A4 substrate, KCZ can either induce hepatic toxicity through increased plasma concentrations or, if used appropriately, can increase compound efficacy. For example, KCZ increases the bioavailability of docetaxel using a lower SOC dose in breast cancer patients [60]. In our case, BZA is not metabolized by CYP3A4. If KCZ is combined with other GP130 inhibitors, hepatic toxicity should be closely monitored, or KCZ should be substituted with a KCZ derivative that retains tGLI1-selectivity without CYP3A4 inhibitory effects.

There are currently no FDA-approved IL-6/IL-6R/GP130 inhibitors for the treatment of breast cancer. However, there are many approaches to targeting the IL-6/IL-6R/GP130 signaling pathway, such as direct inhibition of IL-6, IL-6Rα, GP130, JAK2, and STAT3. Originally FDA-approved as a SERM for post-menopausal osteoporosis, BZA has been clinically investigated for its estrogen-modulatory effects as a hormonal-based therapy for high-risk patients of breast cancer [66]. BZA reduces high-risk biomarkers and provides clinical benefit for hormone receptor (HR)+/HER2- breast cancer patients when combined with CDK4/6 inhibitor palbociclib (NCT02729701, NCT02448771) [66,67]. Interestingly, BZA exhibited preclinical efficacy against HR-breast cancer, which was later attributed to its ability to bind the D1 domain of the GP130 receptor and subsequently disrupt IL-6/IL-6R/GP130 signaling [32,68].

To date, two IL-6Rα monoclonal antibodies (mAbs), a JAK1/2 inhibitor, and a small-molecule STAT3 inhibitor are being clinically investigated for breast cancer. Tocilizumab, an IL-6Rα mAb originally FDA-approved for rheumatoid arthritis, is being investigated in combination with trastuzumab and pertuzumab for the treatment of metastatic trastuzumab-resistant HER2-enriched breast cancer (NCT03135171). Additionally, tocilizumab is also currently under investigation as an immunotherapy-based combination regimen for metastatic TNBC patients (NCT03424005). IL-6Rα mAb, sarilumab, is also FDA-approved for rheumatoid arthritis and is under clinical investigation in combination with capecitabine for the treatment of metastatic TNBC (NCT04333706). Despite recent clinical advancements with anti-IL-6Rα antibodies in breast cancer, mAbs are large, lack BBB permeability, are expensive to develop, and, therefore, are not the best approach for our combination treatments.

The JAK1/2 inhibitor, ruxolitinib, is FDA-approved for the treatment of myelofibrosis and has been extensively tested in breast cancer patients. Ruxolitinib was tested as a single agent in pSTAT3-positive TNBC patients yet did not reach the efficacy endpoint, suggesting alternative mechanisms may be driving therapeutic resistance (NCT01562873). Additional clinical trials are investigating ruxolitinib in combination with chemotherapeutic or immunotherapeutic agents to overcome resistance in TNBC patients (NCT03012230; NCT02928978). Alternative JAK2 inhibitors, fedratinib and pacritinib, are currently under clinical investigation with patients harboring solid tumor types, including within the breast (NCT01836705 and NCT04520269). STAT3 inhibitor, TTI-101, recently gained a fast-track designation from the FDA for the treatment of hepatocellular carcinoma (NCT03195699). TTI-101 is also the only STAT3 inhibitor under clinical investigation for advanced breast cancer as well as HR+/HER2-palbociclib-resistant breast cancer (NCT03195699; NCT05384119) and could be a viable candidate to combine with a tGLI1-targeting agent.

## 5. Conclusions

In conclusion, our study reports the following novel findings: (1) Dual targeting of both tGLI1 and IL-6/IL-6R/GP130 signaling pathways synergize to suppress HER2-enriched breast cancer and TNBC in vitro. (2) KCZ+BZA is a novel combination therapy and is efficacious against HER2-enriched breast cancer and TNBC CSC subpopulations. (3) KCZ+BZA combination therapy suppresses TNBC PDX growth as well as TNBC metastatic burden in vivo with limited toxicity. Overall, these preclinical data provide supporting evidence to further investigate dual-targeting tGLI1 and GP130 clinically for metastatic HER2-enriched breast cancer and TNBC patients.

## Data Availability

The raw data supporting the conclusions of this article will be made available by the authors upon reasonable request.

## Supplementary Materials

The following supporting information can be downloaded at: https://www.mdpi.com/article/10.3390/cells13242087/s1, Figure S1: KCZ+BZA reduces CD44^+^/CD24^−^ TNBC cells; Figure S2: Co-treatment with KCZ and BZA is synergistic at a 10:1 and 20:1 ratio in vitro; Figure S3: KCZ+BZA co-inhibition decreases the migratory ability of HER2-enriched breast cancer and TNBC cells; Figure S4: CYP3A4 and AR protein expression in breast cancer cells; Figure S5: Metastasis-free survival (MFS) of Luminal or HER2-enriched breast cancer patients based on tGLI1 and IL-6/IL-6R/GP130 pathway activation; Table S1: RT-qPCR primers.
