# Supplementary material for: Co-Inhibition of tGLI1 and GP130 Using FDA-Approved Ketoconazole and Bazedoxifene Is Synergistic Against the Growth and Metastasis of HER2-Enriched and Triple-Negative Breast Cancers
Source: Cells. 2024 Dec 17;13(24):2087. doi: 10.3390/cells13242087 (PMC11674475; doi:10.3390/cells13242087)

SUPPLEMENTARY DATA

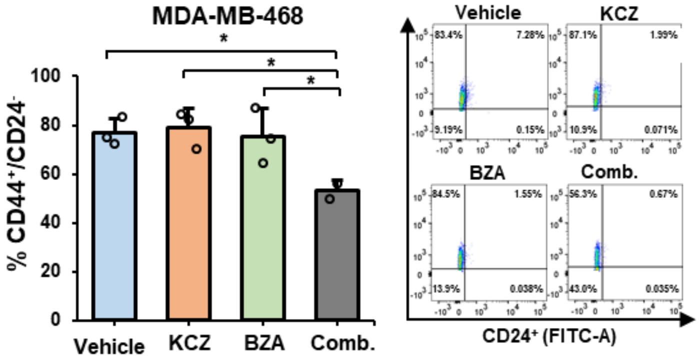

Supplementary Figure S1. KCZ+BZA reduces CD44$^+$/CD24$^-$ TNBC cells. (Ref. Figure 3) Flow cytometry analysis of CD44$^+$/CD24$^-$ TNBC cells MDA-MB-468 post treatment of vehicle, KCZ, BZA, or combination. Note: *, $p < 0.05$; **, $p < 0.01$; ***, $p < 0.001$; ****, $p < 0.0001$; One-way ANOVA with Tukey's multiple comparison post-hoc test was used to compute p-values.

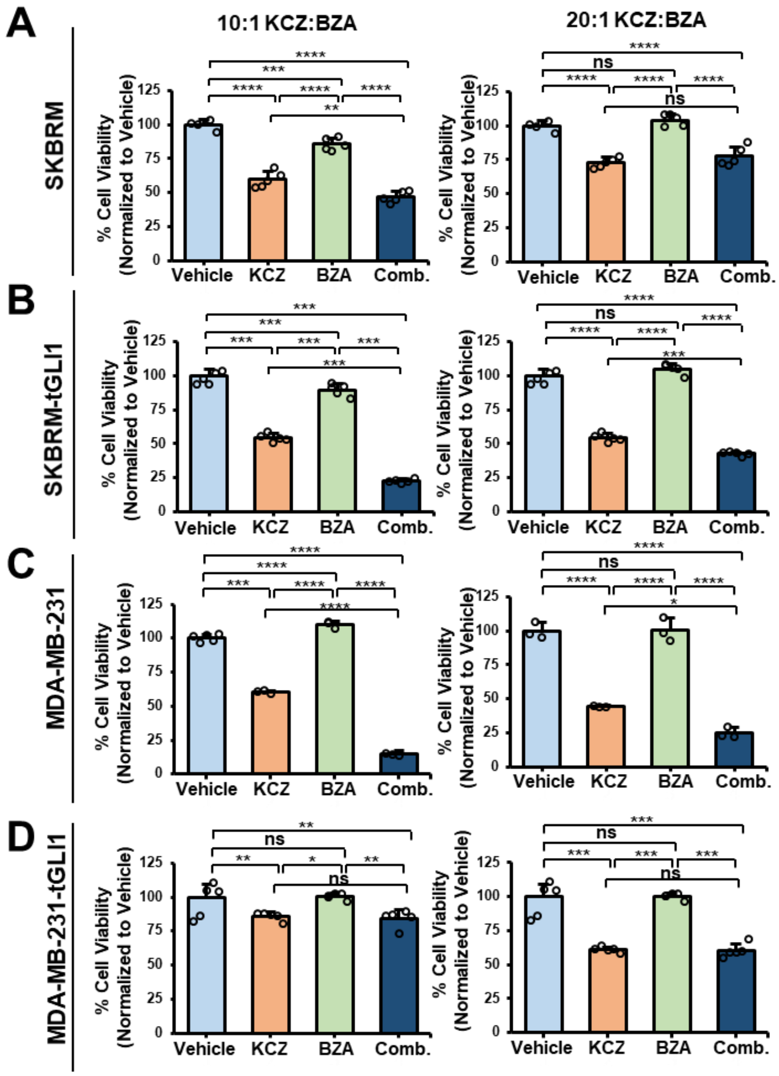

Supplementary Figure S2. Co-treatment with KCZ and BZA is synergistic at a 10:1 and 20:1 ratio *in vitro*. (Ref. Figure 4) SKBRM, SKBRM-tGLI1, MDA-MB-231, and MDA-MB-231-tGLI1 breast cancer cells were seeded in 96-well plates and treated with 5:1, 10:1, or 20:1 ratio of KCZ:BZA for 48 hours. Representative 10:1 and 20:1 ratios of KCZ:BZA viability graphs are shown for SKBRM (**A**), SKRBM-tGLI1 (**B**), MDA-MB-231 (**C**), and MDA-MB-231-tGLI1 (**D**). Note: *, p < 0.05; **, p < 0.01; ***, p < 0.001; ****, p < 0.0001; one-way ANOVAs with post-hoc Tukey's multiple comparison test were used to calculate p-values for all panels.

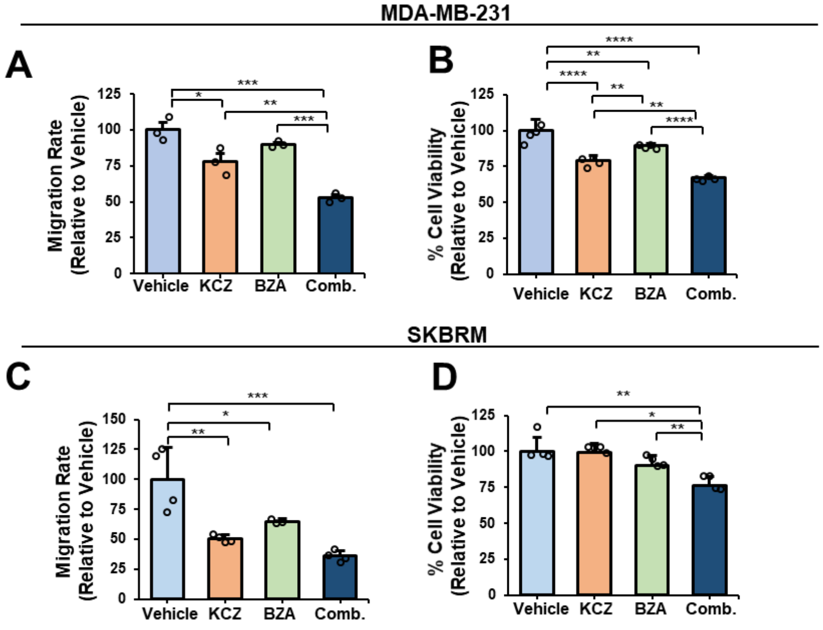

Supplementary Figure S3. KCZ+BZA co-inhibition decreases migratory ability of HER2-enriched breast cancer and TNBC cells. (Ref. Figure. 4) (**A**) Raw migration quantification of SKBRM cells treated with 5:1 ratio of KCZ:BZA for 24 hours. (**B**) Cell viability assay of SKBRM cells to derive a net migration rate. (**C**) 24-hour cell viability of MDA-MB-231 breast cancer cells treated with a 5:1 ratio of KCZ:BZA. (**D**) Cell-based viability assay after 24-hour treatment of vehicle, KCZ, BZA, or combination in MDA-MB-231 breast cancer cells. Note: *, $p < 0.05$; **, $p < 0.01$; ***, $p < 0.001$; ****, $p < 0.0001$; One-way ANOVAs with Tukey's multiple comparison post-hoc test was used to compute p-values for all panels.

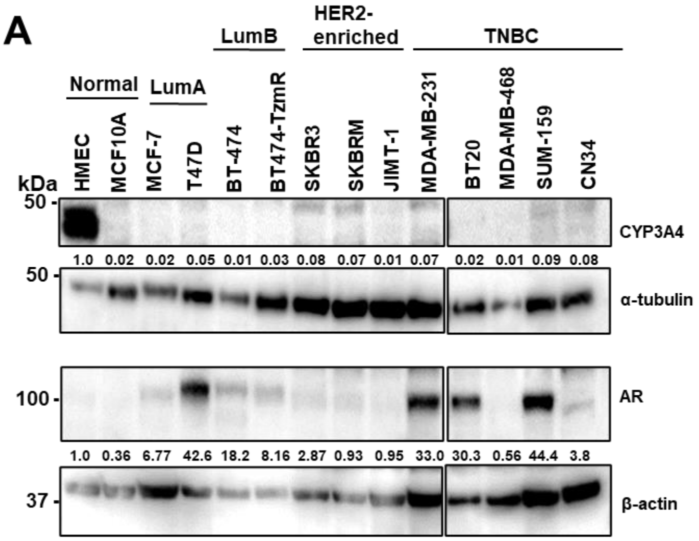

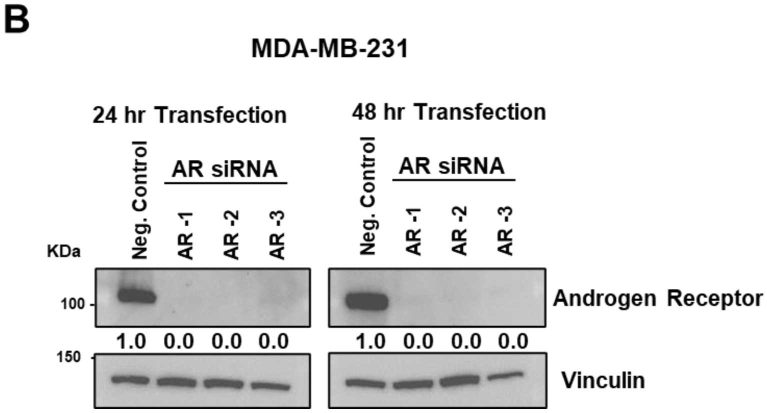

Supplementary Figure S4. CYP3A4 and AR protein expression in breast cancer cells. (Ref. Figure 5) (**A**) Western blot panel of normal mammary epithelial cells and breast cancer cell lines to determine endogenous CYP3A4 (normalized to α-tubulin) and AR protein expression (normalized to β-actin). (**B**) MDA-MB-231 cells were transfected with either negative control (scrambled siRNA) or three different AR siRNAs for knockdown. Cells were transfected for 24 or 48 hours to validate longevity of knockdown. Western blot confirmation of AR expression in MDA-MB-231 cells transfected with negative control or AR siRNA 1-3 (normalized to vinculin). Vinculin serves as a high molecular weight loading control.

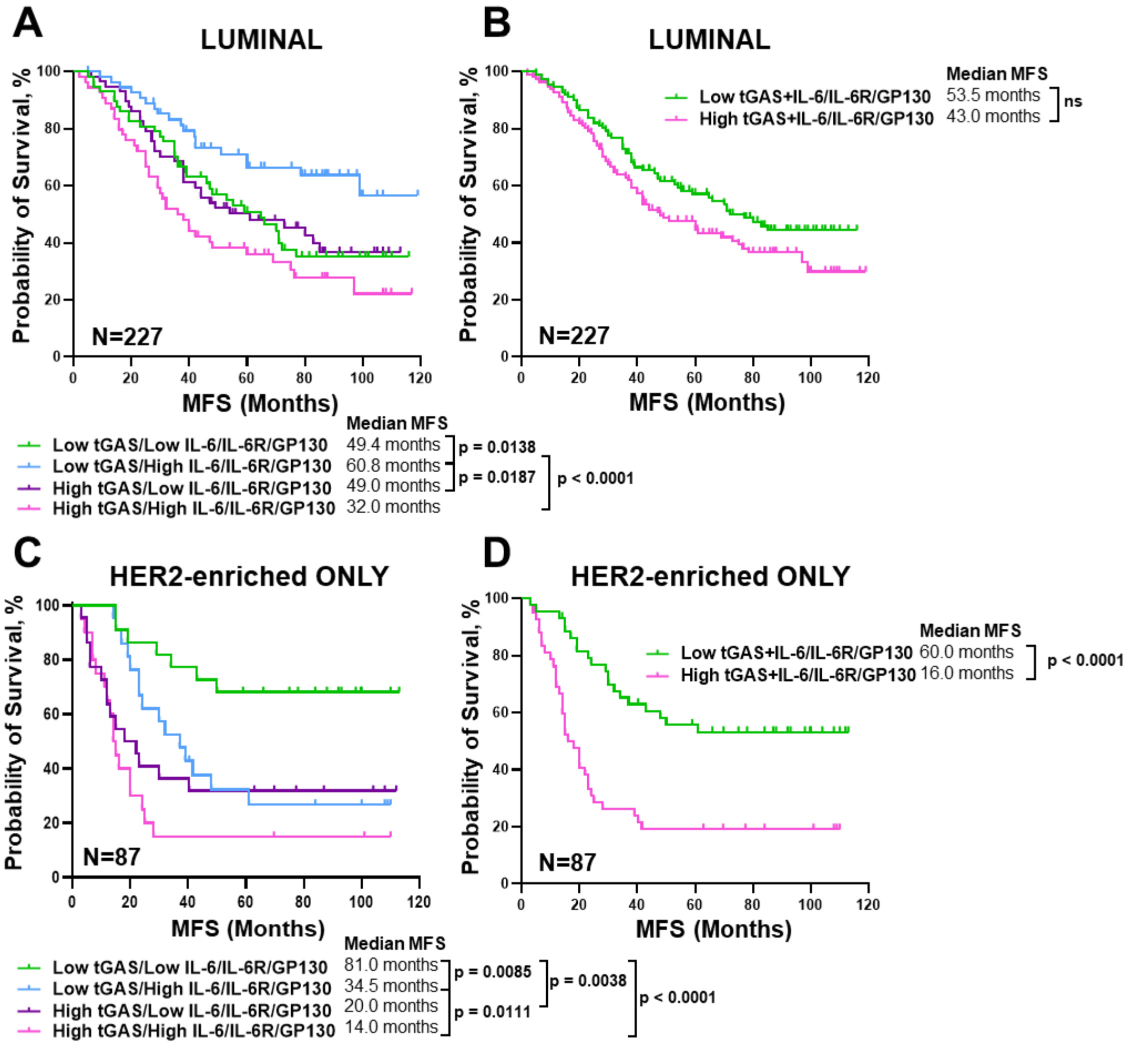

Supplementary Figure S5. Metastasis-free survival (MFS) of Luminal or HER2-enriched breast cancer patients based on tGLI1 and IL-6/IL-6R/GP130 pathway activation. (Ref. Figure 8). (**A**) Kaplan-Meier MFS curve of luminal breast cancer patient cohort stratified into four groups based on the extent of tGAS and IL-6/IL-6R/GP130 pathway activation. (**B**) Combined tGAS+IL-6/IL-6R/GP130 activation on MFS in luminal breast cancer patients (N=227). (**C**) Kaplan-Meier MFS curve of HER2-enriched breast cancer patients based on high versus low tGAS and/or IL-6/IL6R/GP130 pathway activation. (**D**) Combined tGAS+IL-6/IL6R/GP130 activation on MFS of HER2-enriched breast cancer patients (N=87). Kaplan-Meier and Log-rank analyses were used to compute p-values.

Supplementary Table S1. RT-qPCR primers.

Gene	Forward Sequence (5'-3')	Reverse Sequence (5'-3')
tGLI1	GTGTGGGGACAGAAGTCAA	GTGCGGATAACCGTCTGC
GP130	AACAGCATCCAGTGTCACCTT	TCCCCTCGTTCACAATGCAA
Nanog	CTAAGAGGTGGCAGAAAAACA	CTGGTGGTAGGAAGAGTAAAGG
OCT4	TGGTCCGAGTGTGGTTCTGTAA	TGTGCATAGTCGCTGCTTGAT
SOX2	GGAGTTGTCAAGGCAGAGAAGAG	GAGAGAGGCAAACTGGAATC
GAPDH	ACTGCCAACGTGTCAGTGG	GTGTCGCTGTTGAAGTCAGA

Supplemental Figure 1: Uncropped western blot images for Fig. 1A.

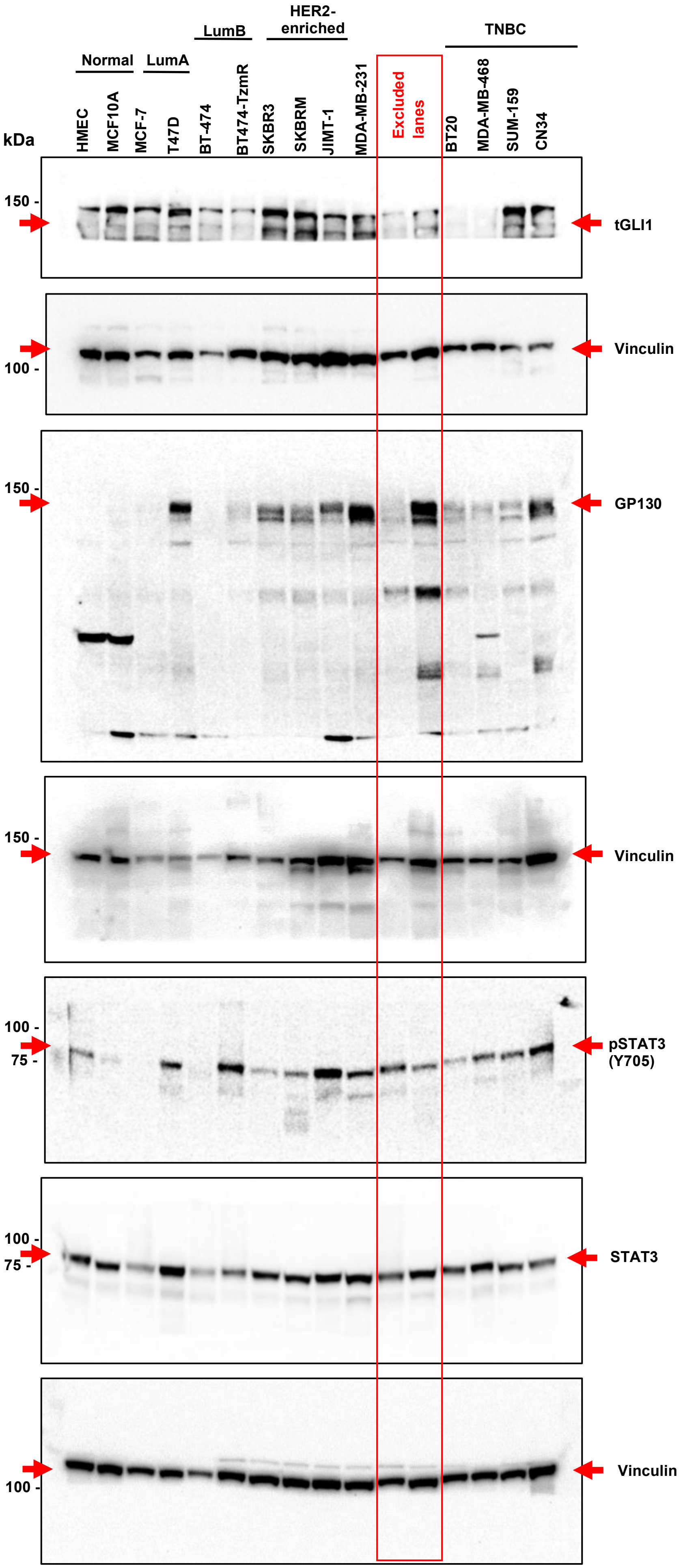

Supplemental Figure 2: Uncropped western blot images for Fig. 2E.

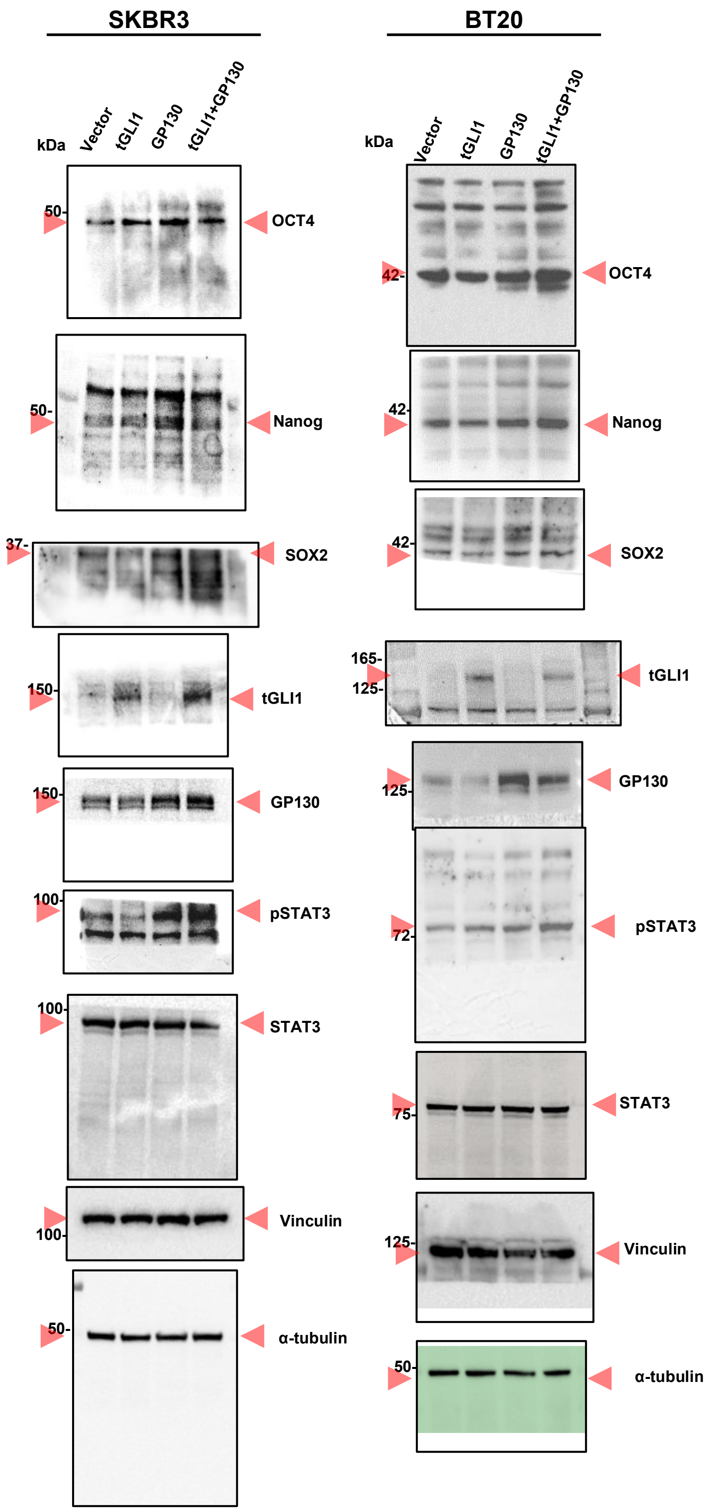

Supplemental Figure 3: Uncropped western blot images for Fig. 3C and Fig. 3E.

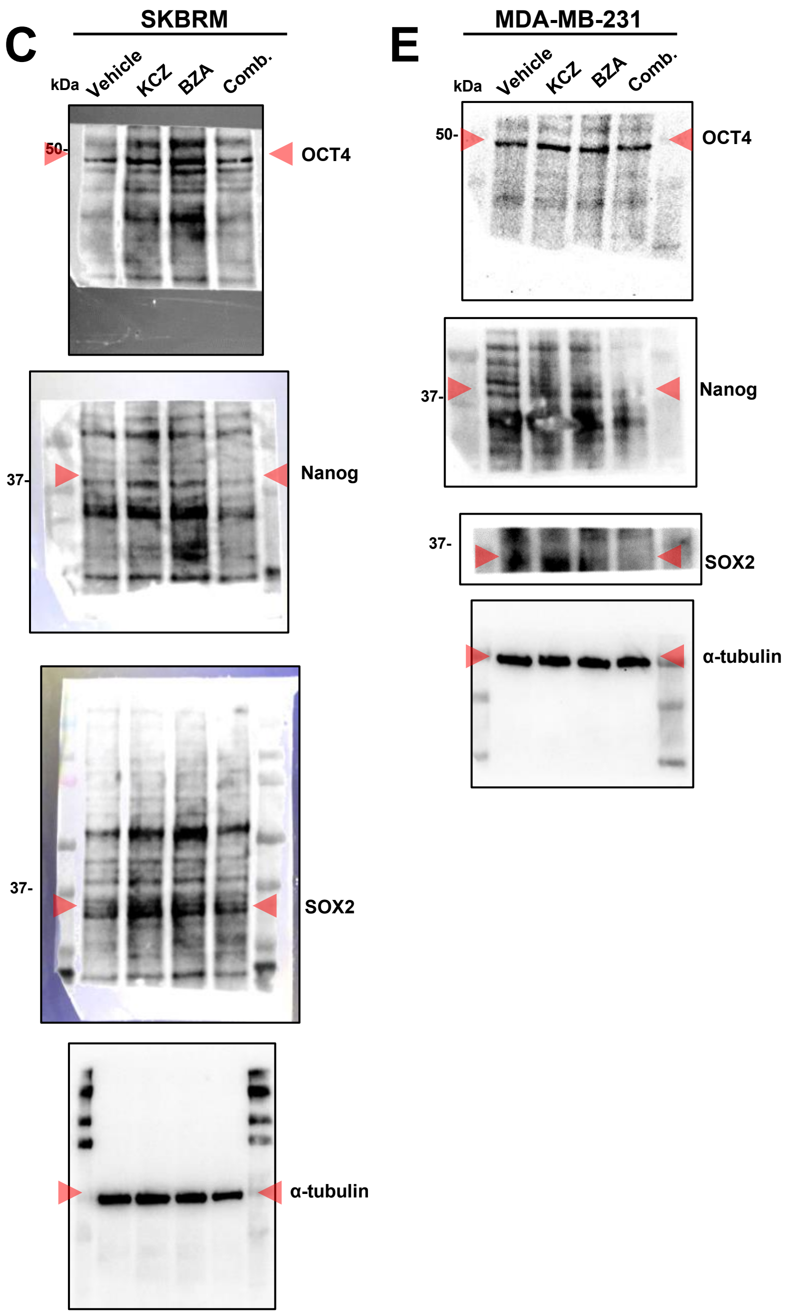

Supplemental Figure 4. Uncropped western blot mages for Fig. 5A-B.

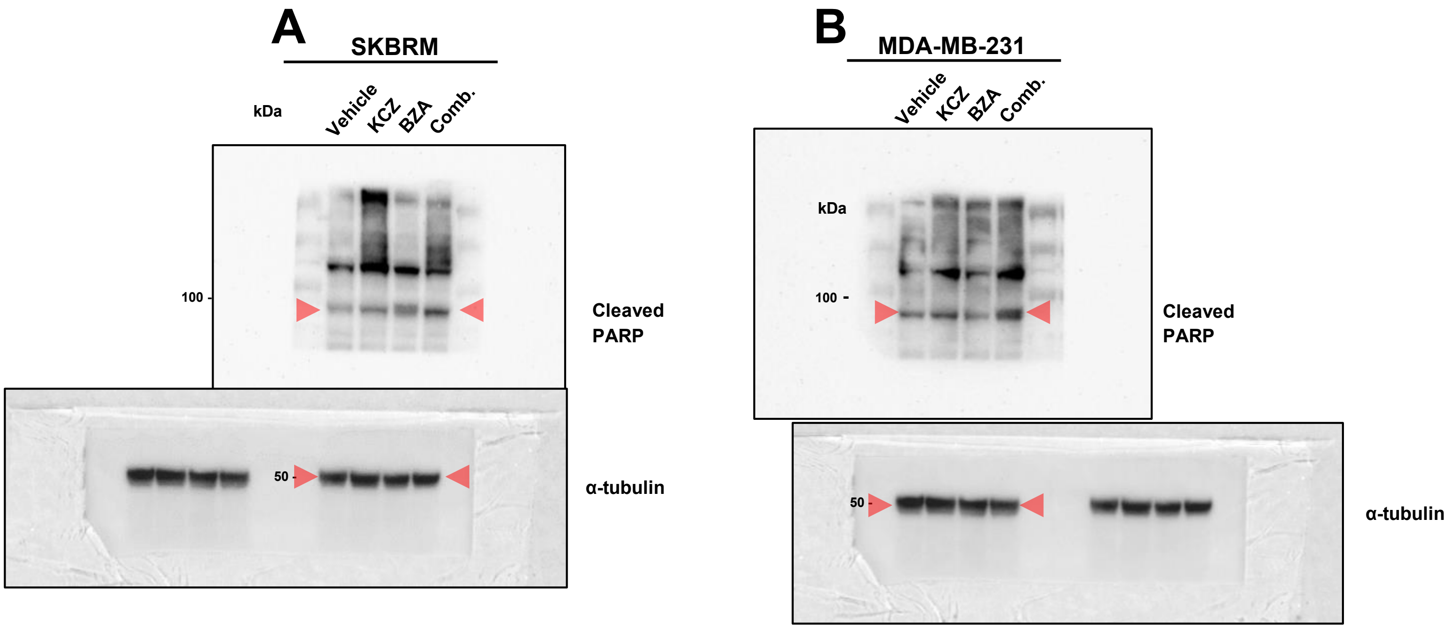

Supplemental Figure 5. Uncropped western blot mages for Fig. 5E-G.

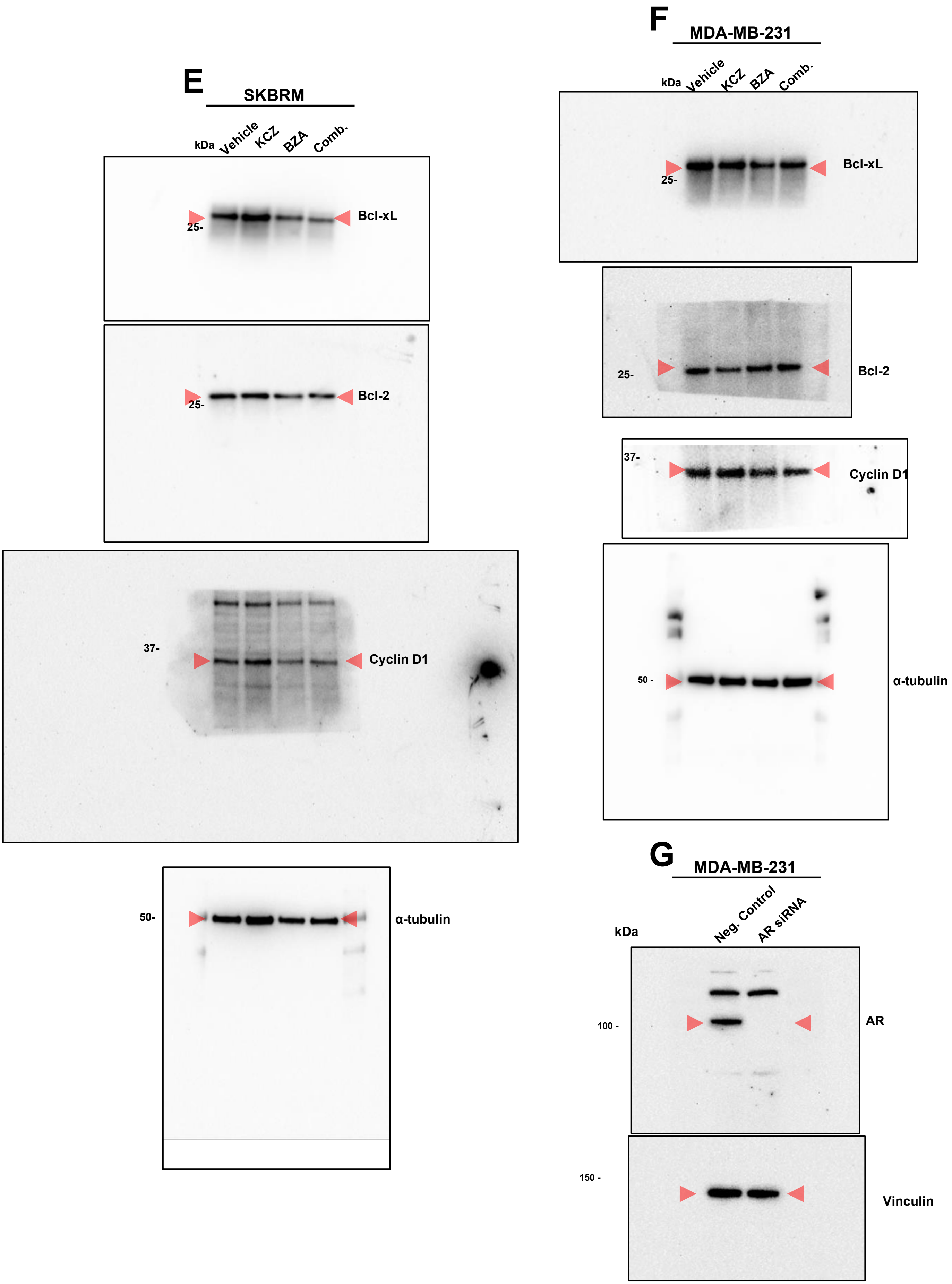

Supplemental Figure 6. Uncropped western blot mages for Fig. 5I-J

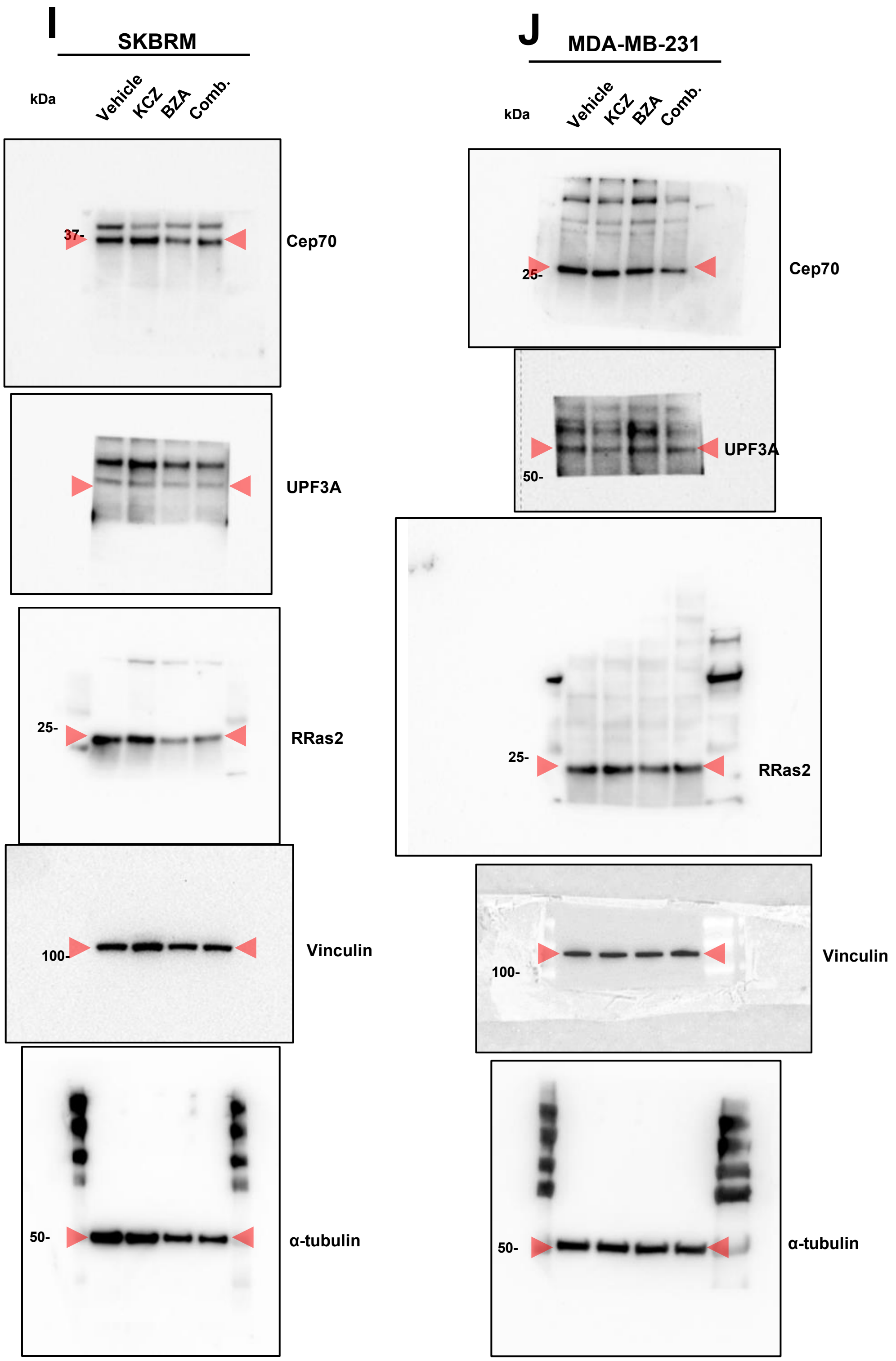

Supplemental Figure 7: Uncropped western blot images for Fig. 6A.

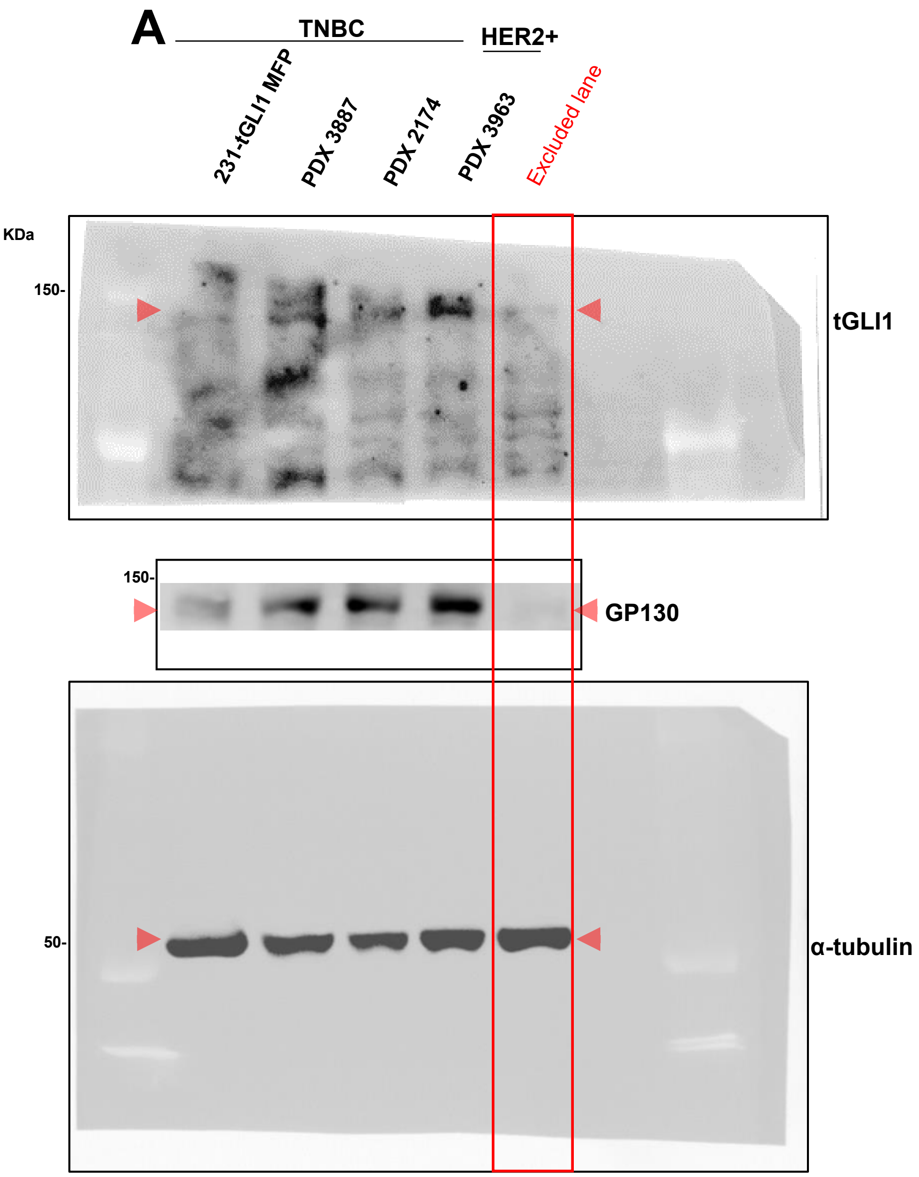

Supplement: Supplementary file 1 [file cells-13-02087-s001.zip › cells-3333392-supplementary.pdf]
